# Waste Reutilization in Polymeric Membrane Fabrication: A New Direction in Membranes for Separation

**DOI:** 10.3390/membranes11100782

**Published:** 2021-10-12

**Authors:** Pei Sean Goh, Mohd Hafiz Dzarfan Othman, Takeshi Matsuura

**Affiliations:** 1 Advanced Membrane Technology Research Centre (AMTEC), School of Chemical and Energy Engineering, Universiti Teknologi Malaysia, Johor Bahru 81310, Malaysia; hafiz@petroleum.utm.my; 2 Department of Chemical and Biological Engineering, University of Ottawa, 161 Louis Pasteur St., Ottawa, ON K1N 6N5, Canada; matsuura@uottawa.ca

**Keywords:** solid waste, polymeric membrane, liquid separation, keratin, cellulose, plastic

## Abstract

In parallel to the rapid growth in economic and social activities, there has been an undesirable increase in environmental degradation due to the massively produced and disposed waste. The need to manage waste in a more innovative manner has become an urgent matter. In response to the call for circular economy, some solid wastes can offer plenty of opportunities to be reutilized as raw materials for the fabrication of functional, high-value products. In the context of solid waste-derived polymeric membrane development, this strategy can pave a way to reduce the consumption of conventional feedstock for the production of synthetic polymers and simultaneously to dampen the negative environmental impacts resulting from the improper management of these solid wastes. The review aims to offer a platform for overviewing the potentials of reutilizing solid waste in liquid separation membrane fabrication by covering the important aspects, including waste pretreatment and raw material extraction, membrane fabrication and characterizations, as well as the separation performance evaluation of the resultant membranes. Three major types of waste-derived polymeric raw materials, namely keratin, cellulose, and plastics, are discussed based on the waste origins, limitations in the waste processing, and their conversion into polymeric membranes. With the promising material properties and viability of processing facilities, recycling and reutilization of waste resources for membrane fabrication are deemed to be a promising strategy that can bring about huge benefits in multiple ways, especially to make a step closer to sustainable and green membrane production.

## 1. Introduction

**Figure 1:**
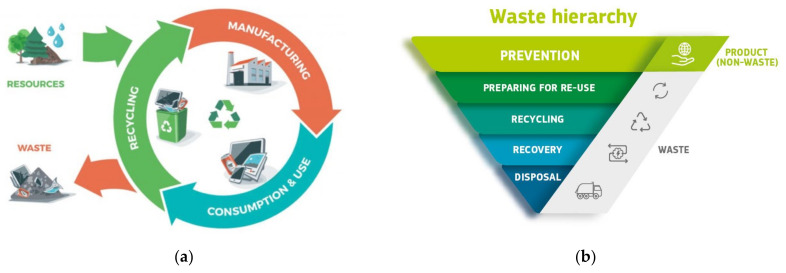
The illustration of the concept of (**a**) circular economy and (**b**) waste hierarchy. Source: Europa.eu (official website of European Union).

Following the dramatically increased industrialization, agricultural and other human activities in parallel with population growth, high consumption, and production have been witnessed in all sectors [1,2,3]. Waste generation is inevitable in many daily activities and industrial processes in which the level of waste generated increases proportionally to the demand for products [4]. Consequently, the challenges associated with resource management and the handling of waste are also rising at a rapid pace. The improper disposal and management not only lead to major environmental concern but also impose negative impacts on human health [5,6,7]. The extreme scale of such issues obliges researchers to seek solutions that include the use of more sustainable resources and establishment of cleaner technologies. The alarming level of waste generation has urged more studies and research to focus on approaches related to waste management, product life cycle assessment, and circular economy [8,9,10]. As depicted in Figure 1a, the circular economy is a resource-efficient model that emphasizes eliminating waste and making the entire economy more sustainable through waste reuse, reduce, and recycling. For economic and environmental concerns as well as to comply with the newly established reinforcements on green economy, circular strategies have been proposed in industrial sectors to realize the recovery of waste products that have potentials to be used as raw materials [11]. Minimizing the use of raw materials and rethinking the source of materials have become the current trend in manufacturing industries. Based on the five-step waste hierarchy sets in the European Union’s Waste Management Directive (Figure 1b), preventing waste is the highest priority, while sending waste to landfill should be the last resort. In response to the needs of generating new recycling opportunities and establishing wider waste-reduction strategy, many wastes that were once regarded as a nuisance in the environment have been favourably converted into useful products. In fact, wastes offer many opportunities to fabricate cost-saving and sustainable materials with comparable or even superior properties to the materials produced from conventionally used precursors. Furthermore, with the depletion of petroleum-based resources and the accelerating environmentally related issues, the utilization of renewable resources becomes an important milestone in material development [12]. The present perspective for the establishment of sustainable industries is associated with the progressive replacement of non-renewable sources by renewable sources.

The circular economy frameworks offer an opportunity for membrane technology innovation. Membrane technology has been applied across a broad spectrum of industrial and municipal installations and is currently popular for water treatment and reuse as well as water desalination, on account of its reliability, high-efficiency, and ease of operation [13,14]. Particularly, membrane-based wastewater treatment technologies are widely accepted in many developed regions to offer high-quality and consistent treatment, to meet the increasingly stringent discharge requirements, and to obtain high-quality discharge that is suitable for beneficial reuse. Liquid separation membrane can be fabricated using polymeric or ceramic materials. The primary advantage of polymeric membranes is that they can be fabricated through well-established techniques and upscaled at a reasonably low cost [15]. Currently, the polymeric membranes commercially used for liquid separation, either in the form of integrally skinned asymmetric membrane or thin film composite (TFC) membrane, are mainly fabricated through phase inversion technique of commercial polymers, such as polysulfone (PSF), polyethersulfone (PES), and cellulose acetate (CA) [16]. Nanofibre membranes fabricated from electrospinning using polymers such as polyacrylonitrile (PAN) and poly(vinylidene fluoride) (PVDF) have also been widely studied for wastewater treatment and desalination [17,18]. Regardless of the type of membrane-separation processes, membrane material selection and dope formulation are critical factors in dictating the membrane-separation performance.

Numerous efforts have been devoted to minimizing the environmental impacts of membrane production. These efforts include reducing the use of chemicals or using green solvents as alternatives to the hazardous solvents. Developing sustainable, bio-based, and biodegradable polymers is another straightforward strategy in achieving this goal [19,20]. While materials scientists are deliberately looking for new synthesis route to synthesize biopolymers, many solid wastes generated from naturally occurring resources offer a readily available source of bio-based and biodegradable polymers [21]. The idea of using waste as a precursor for membranes production can help in curtailing waste disposal to our environment while providing an attractive, low-cost means to utilize fossil-based polymers as membrane material. Moreover, alternatives to conventional polymers derived from fossil fuels are desirable, as the processing of fossil-based polymers is not only related to fossil resource depletion but is also associated to human toxicity, marine ecotoxicity, and global warming due to the volatile emission [22,23]. In view of these threats, there has been surging research interest in using waste-derived polymers, such as cellulose, keratin, chitosan, and rubber, as alternatives for membrane fabrication.

The agricultural industry, which includes all economic activities engaged in plantation crops, farming and mariculture, and forestry, is one of the largest waste-producing industries. Agricultural waste comprises of animal, food, and crop waste that can be commonly found in various agricultural operations around us. It has been an established fact that agricultural waste and forest residues present huge environmental benefits, as they are naturally abundant, renewable, and normally require minimum energy for conversion [24,25]. The application of agricultural waste has been evidenced in several sectors, such as water treatment, biopolymer production, energy generation, food, and pharmaceutical [26]. For instance, agricultural waste has been widely processed into biomass-derived adsorbents for the removal of water-polluting contaminants, such as dyes, heavy metals, and micropollutants [27,28,29,30]. Through biorefineries, biomass has been converted to biofuel and a wide range of high-value pharmaceutical products [31,32,33,34]. Biomass can also serve as a tool in the preparation of construction materials, such as earth block and bricks [35,36]. Other notable wastes are waste polymer and post-consumer plastics. It has been statistically shown that a total of 8.3 billion tonnes of plastic products have been manufactured in the last 60 years. Unfortunately, for the total of 6.3 billion tonnes of plastic waste generated, only 9% of the waste has been recycled [37]. As a low-cost and easily collected post-consumer item, plastic waste can be recycled and converted into many value-added and futuristics products that can be potentially used in energy, fuel, and construction [38,39,40]. Nano and microsized plastics can also be processed into products like media paints and cosmetics. On account of their chemical compositions and properties, both agricultural waste and plastic waste could attractively offer raw materials for polymeric membranes, which represent a high-value option for their upcycling.

In the past few years, the reutilization of industrial and post-consumers wastes to obtain high-value liquid separation membrane has been increasingly investigated. These studies have evidenced the potential of a wide range of waste to be recycled as raw materials of polymeric liquid separation membranes. The resultant membranes exhibit separation efficiency comparable to that exhibited by the membranes fabricated from commercial materials. The potentials of biomass waste, such as cellulose, lignin, and chitin, to be reutilized for the preparation of various functional polymeric materials were revealed by Wang et al [41]. In their encyclopaedia article, Maiti and Pandey provided an overview on the recycling process of waste polymers, including polyethylene (PE) and polypropylene (PP) for membrane fabrication, highlighting this approach as a promising effort towards circular economy in membrane manufacturing [42].

**Figure 2:**
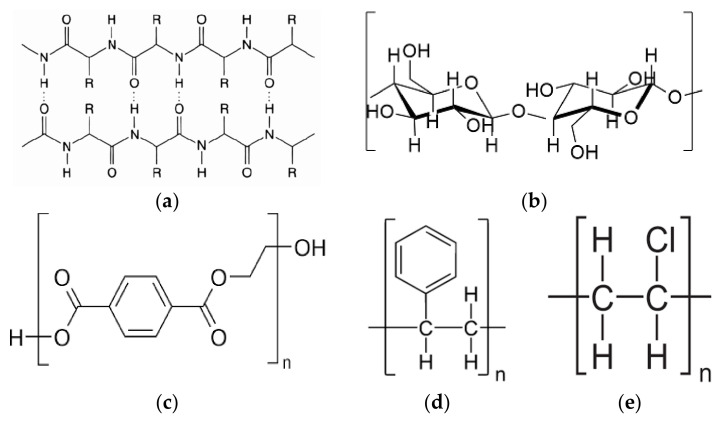
Chemical structure of (**a**) beta-keratin, (**b**) cellulose, (**c**) polyethylene terephthalate, (**d**) polystyrene, and (**e**) polyvinyl chloride.

In response to the wide positive impact and benefits, the present review aims to advance the understanding and development of this important area by looking at the potential waste sources for polymeric membrane fabrication, giving particular attention to their physicochemical properties and bottlenecks in recycling or recovery. Three classes of raw materials, i.e., keratin, cellulose, and plastics, as structurally illustrated in Figure 2, are discussed in this review. These materials can be widely found in domestic/industrial solid wastes and can be potentially used for liquid separation membrane fabrication. Their origins and sources, dissolution, and extraction methods are first discussed to provide an overview on their availability and requirements to be used as a raw material for membrane fabrication. The membrane fabrication methodologies, which include the processing of waste for raw material extraction and dissolution, as well as the resultant membrane properties and functions are presented based on the neat regenerated/recycled materials and their composites. The challenges in solid waste processing for their transformation into polymeric liquid separation membranes are also highlighted with some suggestions and future research directions to close the current gaps. It is hoped that this review can motivate and open a new avenue for exploring solid waste as a sustainable source of polymeric raw materials as well as to fetch more attention not only from the scientific community but also from membrane manufacturers to stimulate their interest in converting these highly potential waste materials into high-value products.

## 2. Potential Waste Sources for Polymeric Membrane Fabrication

### 2.1. Keratin

Keratin is a fibrous, structural protein commonly found in wool, hair, and poultry feather [43,44]. Wools obtained from textile fabric and hairy parts of animals are especially an important keratin extraction sources, as it consists of ~95% raw keratin [45]. Chicken feathers, which consists of ~90% protein keratin, are another major source for extracting keratin [46]. The living-organism leftovers produced in tanning, slaughterhouse, and poultry industries are treated as waste, and their disposal often leads to vast environmental problem. A total of 90% of the 1.2 million tons of raw wool produced annually has been processed in the textile industry [47]. Similarly, the chicken feathers produced as by-product of the poultry industry are mainly disposed through landfills; only a small quantity is pretreated and used as low-nutritional-value animal feed or other low-grade products [48]. The combined annual generation of keratin waste associated with wool and feathers has been estimated as 30 billion tons [49]. The valorisation of these wastes to obtain sustainable source of keratin will reduce the issues of residue disposal and offer a solution for circular economy [50]. Keratin extracted from wool or hair can be formed into various forms and shapes, such as film and coating [51], scaffold [52], hydrogel [53], and nanofibre. Keratin has very wide application, ranging from biomedical and water remediation to food packaging and cosmetics, owing to their biocompatibility, biodegradability, absorptivity, and antibacterial properties [54,55,56].

Keratin is made from polypeptide with tight packing secondary structure and can self-assemble into a network of strands that are crosslinked through disulphide (S-S) bonds. Significant functional differences exist depending on the nature and compositions of the keratins found in different sources; hence, this factor must be taken into consideration when reutilizing keratin for the production of new functional materials [57]. The keratins of feather and wool have different composition and morphological structure. For instance, chicken feather keratin mainly consists of β-sheet structure and a high percentage of amino acids [58]. The total solubilization of wool keratin is more difficult due to the higher cysteine content in the range of 15.9–12.7 g/100 g protein, which is nearly double of that of feather keratin [59]. The molecular weight distribution of feather keratin, with an average of 10 kDa, is also relatively narrow compared that of wool keratin. By using different sources of keratin for hydrogel formation, it has been observed that the low molecular weight and β-sheet conformation of feather keratin promoted hydrogel self-assembly to form rigid structure, while the high molecular weight and α-helix conformation of wool keratins resulted in more flexible hydrogels [60].

Due to its highly stable crosslinked inter- and intra-chains and tight packing of α-helix and β-sheet structure, keratin only becomes soluble under controlled conditions. The commonly used protein dissolution methods are normally ineffective for the solubilization of keratin. To overcome the disulfide bonds, the dissolution and extraction of keratin can be accomplished using oxidative and reductive chemistries [61], ultrasonic treatment [62], and alkali hydrolysis. Extraction of keratin has also been accomplished using ionic liquids (ILs) [63,64] and deep eutectic solvents (DES) [65,66] with powerful dissolving properties. Xie et al. reported the first attempt of using 1-butyl-3-methylimidazolium chloride IL to directly extract keratin from wool fibres, where solution concentration up to 10% (*w/w*) was obtained [67]. From an economic point of view, percarbonate oxidation and sulfide reduction have been suggested as the most feasible technique for scaled-up wool keratin solubilization for application in the textile industry [68]. Using sodium thiosulphate solution, 75.3% of keratin with a molecular mass less than 31 kDa was successfully extracted from a coarse wool source [69]. More than 80% yield of soluble chicken feather keratin was achieved in its extraction by a mixture of mercaptoethanol and sodium bisulphite [70]. Effective chemical pre-treatment can further enhance the extraction efficiency and increase the yield of keratin [71].

Keratin is made up of various compounds, including cysteine, glutamic acid, and aspartic acid, just to name a few. The functional groups on the molecule chain of keratin, such as hydroxyl, carboxyl, and amino groups, make keratin highly hydrophilic. They also offer the sites for chemical reaction and adsorption for the facile removal of toxic metal ions and organic substances [72,73,74,75]. The nanofibre membrane fabricated by mixing keratin and hydrophilic amino acid exhibited both adsorption and filtration capability [76]. Waste-derived keratin nanofibres have been used for membrane-based air and liquid filtration [69]. During the preparation of the electrospinning solution, high-molecular-weight polymers are normally added to increase the spinnability and reduce the brittleness of the nanofibres [77]. Keratin-containing membrane demonstrated excellent dye-removal efficiency owing to its high specific surface area and high porosity.

### 2.2. Cellulose

Cellulose is one of the most abundant biopolymers found in nature. Depending on the sources, lignocellulosic biomass typically contains 40–60% cellulose and 15–30% hemicellulose and 10–25% lignin, which weave to each other to establish a strong-stretch network of recalcitrant biomass [78,79]. Lignin is the main barrier in the deconstruction of lignocellulosic biomass; hence, lignin extraction during biomass pretreatment using solvent is required to ease the conversion of fibrous cellulosic materials [80]. Other sources, such as seaweed, are also gaining attention for cellulose extraction. The removal of lignin through harsh chemical treatment necessary for lignocellulosic biomass is not required for this alternative [81]. Lignocellulosic biomass, like wood, cotton, and flax, are the main sources for industrial-scale cellulose production. Cotton and wood pulp have been generally regarded as the best sources for cellulose extraction owing to their high cellulosic content and relatively easy process to pretreat their lignocellulosic structure [82]. Industrially, cellulose from woody source has been used for the paper production, and natural cellulose fibres of cotton have been widely used for textiles manufacturing. In other words, cellulose can also be recovered from the waste of paper and textile products [83].

Cellulose derivatives and regenerated materials in the forms of films, fibres, and membranes play important roles in several niche commercial areas [84,85]. Cellulose and its derivative are excellent membrane materials and have a long history for membrane fabrication owing to its hydrophilicity, biodegradability, stability, and recyclability. Cellulose acetate (CA), obtained from the esterification of cellulose, is one of the most important commercial cellulose derivatives that have been commonly used for the production of liquid separation membranes [86,87,88]. CA can be readily recycled from a variety consumer and industrial products, including waste cigarette filters, which have been known as one of the most littered and most abundant forms of plastic waste present globally [89,90,91]. Cellulosic membranes can be prepared from commercially available cellulose feedstocks or using natural resources, such as cotton linter [92]. Cellulose and its derivatives have also been introduced as the additive of polymeric materials to improve the properties of the resultant composites. Nanocellulose and microcrystalline cellulose derived from agricultural waste can significantly improve thermal stability and mechanical strength when they are incorporated into the matrices of polymeric films [93]. PVDF membrane incorporated with cellulose nanocrystal, a cellulose derivative obtained from the acid hydrolysis of cellulose, exhibited improved water flux by 20 times compared to the pristine membrane on account of the hydrophilicity rendered by the cellulose additive [94].

Cellulose can be extracted from diverse waste sources. Agricultural wastes and woody residues are known as important sources of cellulose. Oil palm residue is one of the most abundant plantation wastes in palm-oil-producing countries, like Indonesia and Malaysia [95]. Oil palm waste can also serve as a promising cellulose source [96]. Up to 40% *w/w* of cellulose has been successfully extracted from oil palm fronds [97]. Nanocrystalline cellulose extracted from oil palm empty fruit bunch has high adsorptive capacity for the remediation of industrial contaminant [98,99]. The continuous growth of chemical- and water-intensive textile industry results in significant production of waste. As the most important raw material after synthetic fibres, the quantity of cotton waste generated either during pre-consumer stage, such as yarn, fabric, and garment product manufacturing, or during post-consumer stage, where the waste is disposed upon the end of product service life, also rises accordingly. Consisting of >95% cellulose in its composition [100], cotton textile can be potentially processed to obtain cellulose when the appropriate solvent is identified. Every year, approximately 400 million tons of waste paper, including office papers, newspapers, and cardboards, are generated, becoming one of the major contributors of municipal and industrial waste [101]. Waste newspapers, which contain a large amount of cellulose, lignin, and hemicellulose, have been regenerated to form translucent or transparent film with high mechanical strength [102]. Besides recycling waste paper into low-value materials, such as newspaper and boxboard, the conversion of these wastes into high-value cellulose-based products offers more commercially attractive options. Cellulose-based membrane, which has played important roles in various applications, is known as one of such products [103].

The major challenge in cellulosic chemicals or more specifically cellulosic membrane preparation is the limited number of solvents available for cellulose dissolution due to the strong inter- and intra-molecular hydrogen bonding and the partial crystalline structure present in the macromolecule [84,104,105]. NaOH/urea [106,107,108] or NaOH/thiourea solvent mixture has been used to dissolve a small amount of cellulose directly to produce a satisfactory amount of cellulose with mass fraction typically in the range of 4–14%. The dissolution mechanisms of cellulose in these systems have also been investigated, but some controversies regarding the roles and interactions of the alkali and urea during the dissolution process still remain [109,110,111]. IL and IL-cosolvent systems, such as the mixture of 1-ethyl-3-methylimidazole acetate ((Emim)(OAc)) and dimethylsulfoxide (DMSO), allow the preparation of cellulose dope solution without requiring any pre-treatment or activation steps [112,113,114]. Particularly, imidazolium-based ILs have been recognized for their good ability to dissolve cellulose [115]. Glinska et al. compared the efficiency of tetraalkylphosphonium-based and imidazolium-based IL in extracting cellulose from corn stover [116]. While both ILs were able to break the cell wall and dissolve the cellulose component to obtain up to 84% of cellulose yield, microemulsion-forming imidazolium ILs exhibited higher efficiency in removing ash from corn stover and could be more easily recycled compared to the phosphonium counterparts.

### 2.3. Plastics and Rubber

Plastic products are ubiquitous and almost indispensable in everyday lives. With their attractive properties and wide applications in almost all commercial sectors and household activities, the plastic industry has flourished for decades on a large scale [117]. To date, plastic production has approximately reached 8300 million metric tons, and packaging is the largest constituent in the plastic commercial market [118]. The increase in plastic production and consumption comes along with serious environmental challenges. The monomers of commonly used plastics are majorly derived from non-renewable fossil hydrocarbons, so these plastics are highly durable and generally not biodegradable. The share of plastics in municipal solid waste has increased tremendously. Nearly 80% of plastic debris from land-based sources ends up in the ocean as a result of mismanagement of the plastic waste throughout the plastic supply-chain life cycle, starting from production until post-consumer disposal [119]. The non-biodegradable nature of most commercially used plastics lead to their landfill accumulations for many years, hence resulting in direct plastic contamination [120]. On the other hand, the incomplete disintegration of durable plastics result in the formation of microplastic polymer particles of various morphologies that invade the environments and food chains. Therefore, efforts to recycle post-consumer plastics, such as polyethylene terephthalate (PET) bottles, which have been used as single-use packaging for beverages, are necessary to counter white pollution and the subsequent issues resulted from their disposal.

Plastic materials, such as PET [121,122,123], high-density polyethylene (HDPE), polyvinyl chloride (PVC) [124,125,126], and high-impact polystyrene (HIPS) [127], have been used for polymeric membrane preparation. Besides being light-weighted, their high mechanical strength and good chemical resistance against organic solvents as well as inorganic acids and alkali also prompted their applications as membrane materials. Due to the wide availability of these plastics as post-consumer waste as well as their ease in processability and film-forming ability, these recyclable plastics are potential materials for membrane fabrication. The upcycling of plastic waste into polymeric membrane requires less tedious procedure than other sources, such as biomass-derived raw materials, for which pretreament, dissolution, and extraction are necessary to obtain the desired component. By using the same solvent and preparation protocol as their commercial counterpart, plastic waste can be readily converted into membrane of various configurations. However, like other conventional hydrophobic high performance thermoplastics, the hydrophobic nature of most waste-derived plastics has become the major challenge in their reutilization as raw materials for liquid separation membrane [128]. The blending of plastic waste with hydrophilic polymers or additives can overcome this limitation. Commonly used hydrophilic additives, such as polyvinylpyrrolidone (PVP) and polyethylene glycol (PEG), can be suitably used to improve the hydrophilicity as well as the mechanical strength and porosity of the plastic-derived polymers [129,130,131]. The hydrophobic nature of most plastic materials could be an ideal candidate for membrane distillation, which relies on a microporous hydrophobic membrane to prevent mass transfer of liquid, thereby creating a gas–liquid interface.

With the on-going demand and use of rubber, especially in automotive industries, post-customer tyres, which are normally stored in stockpiles or recycled in landfills for road making, can serve as a source of recycled material for polymeric membrane production [132]. Tyre recycling has been commenced for decades, and the recycling rate has become more intense with more advanced strategies used in tyre waste management and recycling, especially in the bigger tyre production regions, such as Europe and the United States. Through physical processes, such as ultrasonic and microwave methods, or chemical processes, such as pyrolysis and chemical dissolution [133], the reclaimed tyres are converted from crosslinking rubber to amorphous polymer, where the three-dimensional structure is also transformed to one-dimensional through the destruction of rubber/sulfur bond in the devulcanization process. However, with more new materials or additives brought in the composition of many rubber products particularly in tyre, the reutilization becomes more challenging.

## 3. Development and Performances of Waste-derived Polymeric Membranes for Liquid Separation

### 3.1. Keratin

**Figure 3:**
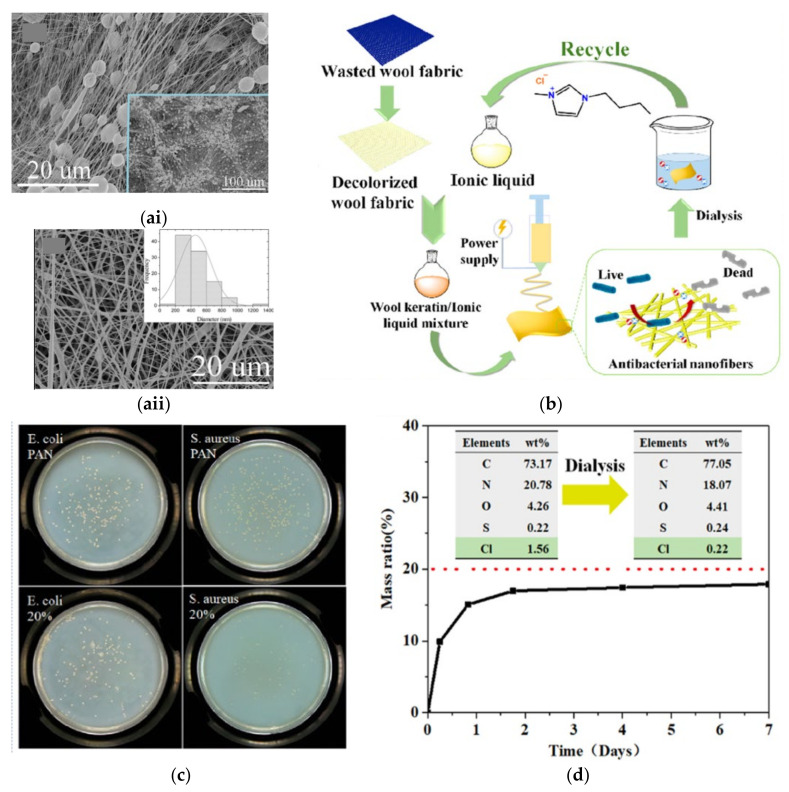
(**a**) SEM images of (i) keratin and (ii) keratin/PEO nanofibre membrane [134]. (**b**) Schematic diagram of the preparation of antibacterial nanofibre recyclable ((Bmim)Cl) IL as solvent. (**c**) Antibacterial test of PAN and PAN/keratin using *S. aureus* and *E. coli* colony, (**d**) weight loss ratio curve, and the elements distribution after dialysis of PAN/keratin for IL solvent recycling [136].

Decolourization of dyed wool fabric is a necessary pretreatment prior to keratin extraction and dissolution. Decolourization of dyed wool fabric was performed through a catalytic reaction using ironphthalocyanine and H_2_O_2_ [134]. The decolourization efficiency increased with the elevated pH, ironphthalocyanine concentration, and H_2_O_2_ concentration. Ironphthalocyanine with high structure stability could break the azo dyes into colourless products and achieve decolorization percentage of ~94.2% when coupled with H_2_O_2_ under catalytic oxidation at pH 10. The decolourized wool fabric was dissolved in formic acid in which poly(ethylene oxide) (PEO) was introduced into the electrospinning solution to improve the spinnability of the wool keratin. The nanofibre membranes were obtained through electrospinning using a voltage of 14 kV, a syringe-collector distance of 15 cm, and rate of 0.5 mL/h. As illustrated in Figure 3a, with the addition of 10 wt% PEO, bead formation was suppressed, and the average diameter was increased from 226 ± 112 nm to 379 ± 226 nm. A solution containing feather-derived keratin, PEO, and PVA was electrospun, followed by crosslinking with citric acid and glyoxal vapor to obtain NF membranes that can be suitably used for solvent-resistant filtration [135]. The keratin content was extracted from chicken feather using peracetic acid solution and precipitated through dialysis. The average diameter of the nanofibres increased from ~223 nm for pristine nanofibre membrane to ~342 nm and ~304 nm for the citric- and glyoxal-crosslinked membranes, respectively, due to the diffusion of the crosslinked chains into the macromolecular chains to form the crosslinking network. The crosslinking effectively enhanced the intermolecular interaction of composite membranes, which in turn contributed to improved mechanical strength and thermal stability.

Wool fabric keratin extracted with 1-butyl-3-methylimidazolium chloride ((Bmim)Cl) IL was blended with PAN for the preparation of NF membranes through electrospinning [136]. Figure 3b schematically illustrates the procedure of keratin/PAN nanofibre membrane. The protein secondary structures experienced chemical changes upon the dissolution of keratinous materials in IL. The presence of IL solvent with high ion conductivity in the spinning dope promoted the formation of thinner, more uniform, and bead-free nanofibres. In addition, the IL, which consists of imidazole cation and chloride anion, shares the same structure as quaternary ammonium salt to endow antibacterial property. The electrostatic force between the positively charged N atom in the IL cation and negatively charged cell membrane phospholipid bilayer resulted in cell membrane rupture and bacteria death. Compared to PAN membrane with no anti-bacterial activity, the PAN/keratin nanofibre membrane exhibited good antibacterial properties, with 89.21% and 60.70% inhibition rate against E. coli and S. aureus, respectively, as indicated by the bacteria colony count shown in Figure 3c. The moisture-management testing results revealed that the PAN/keratin membrane demonstrated improved wetting and moisture transport properties compared to that of PAN membrane owing to the presence of hydrophilic functional groups of keratin. After several cycles of filtrations, the IL solvent used for the dissolution of wool keratin was recycled through water immersion of the membrane to allow the release of IL from the membrane. The elemental analysis (Figure 3d) revealed that dialysis was an effective approach to recycle IL, as it achieved half balance in the first six hours and almost complete balance after seven days, where almost 90% of IL has been recovered. Pure IL could be obtained through the subsequent rotary evaporation separation and used for the dissolution of a new batch of wool fabric.

A nanofibre membrane for dye removal has been fabricated by electrospinning the polymer dope prepared from the blending of commercial polyamide and keratin extracted from waste goat hair [137]. The keratin was extracted through sulphitolysis, and the keratin solution was prepared by dissolving the hydrolysed keratin in formic acid. Nanofibre membrane with fine, randomly oriented, beadles, interconnected pore structures were then obtained by electrospinning the polymer dope at voltage of 20 kV at a distance of 22 cm and rate of 0.1 ml/h. Compared to the nanofibre with only polyamide, polyamide/keratin nanofibre membrane was characterized by smaller mean pore size, higher porosity, and lower crystallinity. The weaker mechanical strength was associated with the hard and rigid structure of the regenerated keratin. The filtration efficiency of the nanofibre membrane with polyamide/keratin was constantly better than the polyamide counterpart when tested for the removal of dark green anionic tannery dye. The optimal dye rejection of 100% was obtained in acidic condition using 100 ppm dye solution. However, the interaction and synergetic effects between polyamide and keratin to complement each other were not given particular attention in this study. Keratin was also extracted from goat hair using zirconium ball-milling technique to allow the conversion of hair into powder [138]. A total of 5 wt% of goat hair-derived keratin was blended with 20 wt% of PSF in a volume ratio of 95:5 to produce a electrospinning dope for NF membrane fabrication. Compared to PSF membrane produced from the same method, the keratin/PSF electrospun membrane exhibited higher surface hydrophilicity and surface roughness. The presence of hydrogen bonding and intermolecular forces between PSF and keratin was responsible for the improved mechanical strength. The keratin/PSF membrane exhibited water flux of 2000 L m^−2^ h^−1^, which was almost two times and four times higher than that of PSF membrane prepared with the same method and commercial PSF membrane, respectively. In the treatment of tannery dye, the dye rejection was 64% and 76% for PSF membrane and keratin/PSF membrane, respectively. The removal efficiencies of other parameters, including chemical oxygen demand and biological oxygen demand, were also higher than those of PSF membrane.

### 3.2. Cellulose and Derivatives

Using oil palm empty fruit bunches (OPEFB), which typically contain 40–50% cellulose as a source of cellulose, Teow et al., prepared cellulose/PVDF membrane for dye wastewater treatment using OPEFB-derived cellulose [139]. The formulation of polymer dope was performed to identify the appropriate solvent and optimal cellulose concentration. Physical cleaning followed by chemical-based dewaxing, delignification, and extraction were accomplished to obtain the insoluble fraction of cellulose as individual strands that were free from hemicelluloses, lignin, pectins, and waxes. By increasing the ratio of cellulose to PVDF in the dope, the hydrophilic cellulose content accelerated the solvent and non-solvent exchange by reducing the dope viscosity and also by weakening the interaction between hydrophobic PVDF with water, hence resulting in membrane with more porous structures. The membrane prepared using N-Methyl-2-pyrrolidone (NMP) solvent also exhibited greater porosity and larger pore size, compared to those prepared from dimethylacetamide (DMAc) due to the higher solubility parameter to accelerate precipitation rate and promote the formation of sponge-like structure. The cellulose membrane with 3 wt% cellulose and cellulose to PVDF ratio of 96:4 prepared using NMP solvent achieved the highest permeate flux of 158.06 L h^−1^ m^−2^ and methylene blue (MB) dye rejection of 34.9% due to its low surface roughness, large pores, and high porosity.

Kapok fibre is an agricultural product obtained from the seed hairs of kapok trees (Ceiba pentandra). Extraction of cellulose nanocrystal followed by its formation into self-assembled cellulose nanocrystal membranes has been accomplished by Mohamed et al. using kapok fibre as the cellulosic source [140]. The cellulose fibres obtained from alkali extraction and acid hydrolysis of kapok fibre were assembled into nanoporous membrane through water suspension casting evaporation technique. The pretreatment increased the cellulose content from 59.6 for raw kapok fibre to 92.8% for the hydrolysed cellulose microfibre, reducing the dimension of micro-scale fibre to form a nanorod or nano-needle structure. The removal of surface wax from the fibre exposed its hydrophilic hydroxyl groups, hence altering its surface wetting property from hydrophobic to hydrophilic. The self-assembled cellulose nanocrystal membrane was characterized by a smooth-surface, hierarchical layering structure and a homogeneous nano-porous structure with porosity of 52.82 ± 7.79%. With the mean pore size of ~21.3 ± 4.12 nm, the membrane can be used for UF. The cellulose nanocrystal membrane exhibited promising removal efficiency of 85% for the removal of 5 ppm MB from a aqueous medium owing to the role of surface hydroxyl groups in promoting adsorption via hydrogen bond or electrostatic interaction.

**Figure 4:**
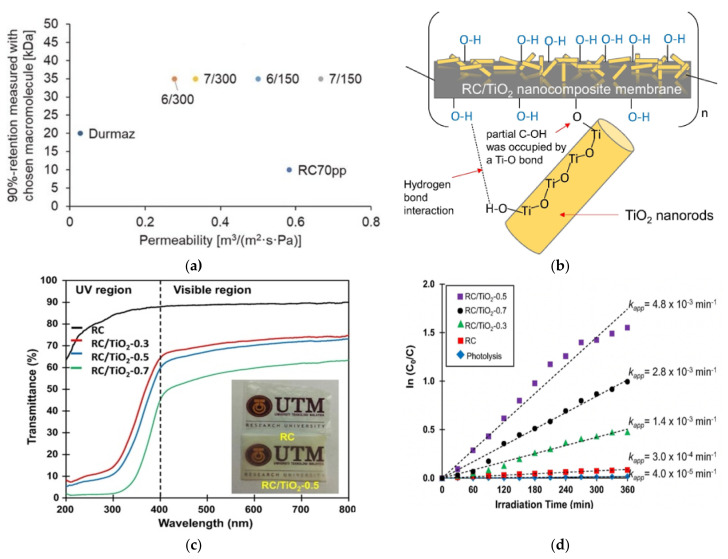
(**a**) Permeability and the retention values of cellulose membranes prepared from waste cotton textile, commercial CA membrane (RC70pp), and cellulose membrane from commercial source (Durmaz) (Sample n/m, n: cellulose wt% m: polymer thickness in µm) [141]. (**b**) Schematic illustration of hydrogen bonding between recycled cellulose and TiO_2_ nanorods, (**c**) optical properties of TiO_2_ nanorods incorporated cellulose photocatalytic membranes, and (**d**) kinetic profile of cellulose photocatalytic membranes incorporated with difference concentration of TiO_2_ nanorods under visible light irradiation [143].

Cellulose obtained from non-dyed cotton bed sheet and dissolved in the mixture of (Emim)(OAc) and DMSO was directly used for UF membrane fabrication without pre-treatment [141]. Cellulose concentration in the range of 5–7 wt% was required to obtain membranes with better adhesion stability and uniform performance. The membranes with 150 µm thickness exhibited PEG 35 kDa retention close to 90%. These membranes possessed similar permeation rate as commercial, regenerated CA membrane and higher permeation rate than cellulose membrane from the commercial source, and the 90% retention was achieved with much larger molecules, as shown in Figure 4a. As the cellulose crystallinity changed during the dissolution and subsequent regeneration processes, less crystalline fraction was found in the membrane matrix compared to that in the cotton textile source. A total of 1 m^2^ of cotton bed linen could be sufficiently used to produce 20 m^2^ membrane with thickness of 150 µm and cellulose concentration of 7 wt%.

Cotton microdust released as waste during spinning process in textile industry has been converted into cellulose membrane through several stages, i.e., acid-alkali heat pretreatment, dissolution in zinc chloride, and finally spin coating, where the cellulose membrane was formed with the simultaneous elimination of lignin using sodium chloride during the coating process [142]. The thermo-chemical pretreatment was effective in removing non-cellulosic components and reducing the microdust agglomeration to facilitate the dissolution in zinc chloride. As a result of the partial removal of lignin, the cellulose content of the membrane increased to 89%, with 8% of lignin in its total composition. The crystallinity of the membrane was significantly reduced due to the transformation of cellulose from its crystalline form to amorphous form during the solubilisation of the cotton microdust with zinc chloride. The direct interaction between Zn^2+^ and -OH group of cellulose was evidenced from the peaks present in the Fourier-transform infrared (FTIR) spectrum. The rheology and tensile studies indicated that the membrane was endowed with good mechanical properties due to the presence of small amount of lignin in the membrane. The assessment of the antibacterial activity of the cotton-microdust-derived cellulose membrane against E. coli revealed that the membrane exhibited satisfactory antibacterial properties due to the incorporation of Zn^2+^ ions during the dissolution process. The cytotoxicity of Zn^2+^ ions induced oxidative stress in the microbial cell, hence retarding the vital cellular functions of the bacterial cell. Although liquid separation efficiency was not evaluated in this study, the physical characterization and antibacterial test suggested that cellulose membrane derived from zinc chloride dissolved cotton microdust can be attractively used for liquid separation owing to its high mechanical strength, anti-biofouling, and hydrophilic feature.

Waste paper can be considered as an abundant but underutilized source of cellulose and its derivatives [144,145,146]. Cellulose was extracted from the non-printed area of recycled newspapers for cellulose membrane fabrication using NaOH/urea as a dissolution medium and H_2_SO_4_ as a coagulant during the phase-inversion process [147]. During the dissolution, NaOH induced ion-pair interactions, which reduced the strong self-association ability of water, establishing hydrogen bonds between urea molecules and cellulose chains. The newly formed intermolecular bond dissolved cellulose and modified its crystalline conformation from cellulose I to II upon contact with the acidic coagulant. The cellulose membrane formed has a homogeneous symmetric dense structure with mean pore size and porosity of 2.48 nm and 41.0%, respectively. During the coagulation using acidic medium with pH values lower than the pKa of cellulosic hydroxyl groups (pKa = 13.4–13.7), the intermolecular interactions in cellulose reduced the anionic nature of the cellulose side chain and the electrostatic repulsion among the adjacent chains, thus resulting in the formation of a dense membrane with low mean pore size. However, due to its structural properties, the membrane achieved unfavourably low pure-water permeability of 0.35 L m^−2^ h^−1^ bar^−1^, suggesting more fine tuning is required to improve the applicability of the cellulose membrane for liquid filtration. In the subsequent work [143], N-doped TiO_2_ nanorod was introduced to the matrix of recycled newspaper-derived cellulose to induce photocatalytic activity in the UF membrane. Homogeneous dispersion of N-doped TiO_2_ nanorod at its low loading was enabled through the formation of hydrogen bonding established through the interaction between the O–H groups of cellulose and Ti–O bond of TiO_2_, as schematically presented in Figure 4b. The incorporation of 0.5 wt% N-doped TiO_2_ improved the membrane porosity from 41% for pristine membrane to 64% due to the increase in free volume between the cellulose chains. Since cellulose could be well dissolved and N-doped TiO_2_ nanorods well dispersed within the polymer matrix, high film transparency (Figure 4c) was ensured, which is known as an important optical property for photocatalytic membrane. The membrane surface hydrophilicity was also increased on account of the superficial hydroxyl group formed on the TiO_2_ surface. This favourably improved the pure water flux (PWF) from 0.69 L m^−2^ h^−1^ for pristine membrane to 4.12 L m^−2^ h^−1^ for the photocatalytic cellulose membrane with 0.5 wt% N-doped TiO_2_ nanorods. The same sample also achieved the highest aqueous phenol degradation kinetic (Figure 4d) and efficiency of 96.6% and 78.8% under ultraviolet and visible light irradiation, respectively.

**Figure 5:**
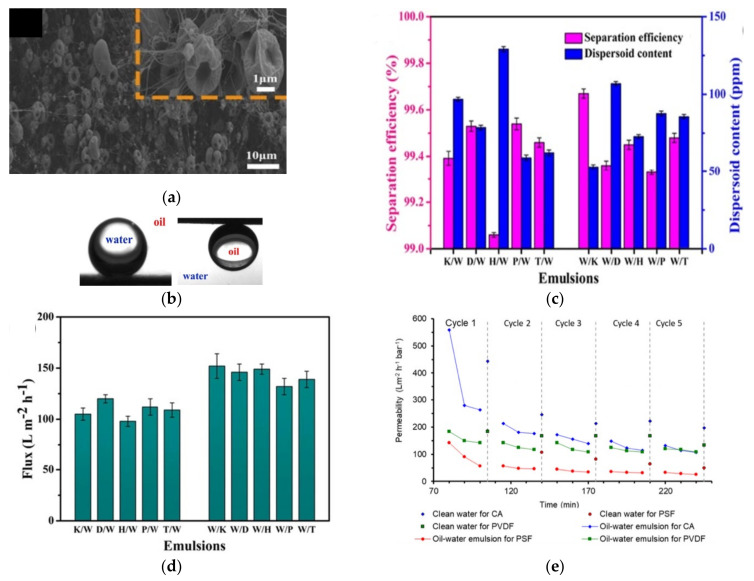
(**a**) Surface morphology of electrospun cigarette filters-derived CA membrane, (**b**) static oil and water droplets showing the superwettability of underwater and underoil, (**c**) separation efficiency and dispersoid content, and (**d**) filtrate flux of various oil/water emulsions (K, kerosene; D, diesel; H, hexane; P, petroleum ether; T, trichloromethane; W, water). [148] (**e**) Oil-in-water emulsion permeability of waste cigarette filter-derived CA membrane, PSF membrane, and PVDF membrane as a function of time for five cycles [149].

CA from waste cigarette filters have been used as raw material for the preparation of electrospun nanofibre membrane to treat oil/water emulsion [148]. After several cleaning cycles, the waste cigarette filter was mixed with dimethylformamide (DMF) and acetone solvent to form the electrospinning dope, which was then electrospun on a stainless steel mesh at room temperature, voltage of 17 kV, and distance between collector and the spinneret of 16 cm. As illustrated in Figure 5a, the surface of the CA nanofibres was characterized by hierarchical micro- or nanoscale roughness and concave beaded on string structure. This structural property is desired to render high underwater superoleophobicity or underoil superhydrophobicity. Due to the surface architecture and superamphiphilic properties, the wettability of the membrane could be tuned by prewetting the membrane with water or oil (Figure 5b) depending on the demand of the oil/water separation. When the membrane was prewetted with heavy oil, it demonstrated underoil superhydrophobicity, in which the oil could easily pass through the membrane while water was retained on another side of the membrane. Using stainless meshes of 2300 mesh size, the electrospun cigarette filters-derived CA membrane exhibited separation efficiency of >99% and low dispersoid content for various oil/water emulsions with filtrate flux of >100 L m^−2^ h^−1^ for both oil-in-water emulsions and water-in-oil emulsions, as shown in Figure 5c,d, in the gravitational driven filtration.

Doyan et al. used waste cigarette filter for the preparation of CA membrane by phase inversion to treat a more challenging oil/water emulsion [149]. Compared with PSF and PVDF membranes fabricated with the same method using commercial raw material, the waste cigarette filter-derived CA membranes exhibited similar morphological structure but higher surface hydrophilicity. The latter feature was rendered by the impurities present in the waste cigarette filter, as can be visually observed from the membrane formed. The membrane exhibited promising permeability of 180 L m^−2^ h^−1^ bar^−1^ after five filtration cycles of oil/water emulsion with rejection of >94.0%. However, despite offering a better oil-in-water emulsion permeability compared with its PSF and the PVDF counterparts as illustrated in Figure 5e, the waste cigarette filter-derived CA membranes suffered from a more severe irreversible fouling of >90.0% due to the stronger oil adhesion on the membrane surface. The findings suggested that, like most CA membranes produced from commercial CA feedstock, optimization of membrane formulation or surface modification of the membrane is required to mitigate the fouling issue.

### 3.3. Polymers and Plastics

Aji et al., demonstrated the feasibility of waste PVC to be utilized as a polymer source for UF membrane [150]. Prior to membrane fabrication through phase-inversion technique, PVC waste collected from post-consumer disposal was dissolved in DMF, and different concentrations of CA were added to overcome the hydrophobicity of PVC. The waste plastic-derived PVC UF membrane achieved BSA rejection of 91%, at par with many other reported PVC membranes prepared from commercial precursors [126,151]. With the increasing CA concentration from 0 to 5 wt%, the PWF and flux recovery ratio increased from 36 L m^−2^ h^−1^ to 85 L m^−2^ h^−1^ and from 56% to 78%, respectively, indicating neat PVC with high hydrophobicity was detrimental to water permeability and antifouling properties. In the subsequent study where gum arabic (GA) was used as an additive in the polymer dope [152], it was revealed that GA not only acted as a pore former like the typical polymeric additive, but it also induced a plasticizing effect for improving the mechanical strength of the PVC membrane. The biopolymer additive enhanced the membrane surface hydrophilicity by 25% compared to that of without additive, consequently improving the water flux from 51 to 98 L m^−2^ h^−1^, with a flux recovery ratio of ~80%. The negatively charged carboxyl group of GA also created electrostatic repulsion with humic acid (HA) molecules, which were used as a model pollutant of natural organic matters. The UF membrane comprised of 15 wt% recycled PVC and 3 wt% GA additive achieved HA removal of 96%, which is comparable or superior to that achieved by UF membranes prepared from commercial PVC with similar concentration but different additives [153,154].

**Figure 6:**
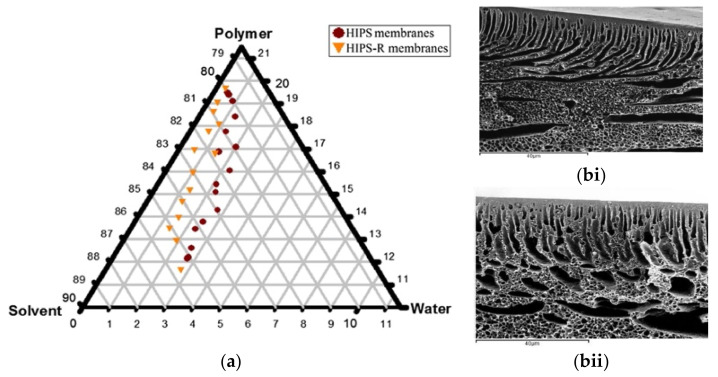
(**a**) Ternary phase diagram constructed based on cloud point measurements with commercial HIPS and recycled HIPS as base polymer and DMF as a solvent. (**b**) Cross-sectional images of membranes formed from (i) commercial HIPS and (ii) recycled HIPS [127].

Recycled HIPS-derived UF membranes was prepared via non-solvent induced phase separation (NIPS) method using DMS as solvent [127]. As demonstrated in Figure 6a, the ternary phase diagrams of HIPS/DMA/water systems constructed by cloud point measurements showed discrepancies of the phase border curves between commercial HIPS and recycled HIPS, i.e., the binodal curve of HIPS-R was shifted toward polymer-water axis, and the homogeneous region was enlarged. The differences were mainly due to the presence of silicon-containing additives introduced during the recycling of HIPS, as verified from the additional peak in the Fourier-transform infrared-attenuated total reflection (FTIR-ATR) spectrum. The recycled HIPS membranes resembled the commercial HIPS membrane in terms of their asymmetric membrane structures but with higher porosity. As a result of lower viscosity of the casting dope, the water in the coagulation bath diffused more rapidly into the cast HIPS-R film, which in turn promoted the formation of macrovoid to form more porous membranes with larger cavities in the sublayer compared to the membranes formed from commercial HIPS (Figure 6b). Owing to the more porous structure and higher hydrophilicity, the recycled HIPS membrane demonstrated higher water permeability. The hydrophilic additive minimized the irreversible fouling, where the mean total fouling resistance (RT) for HIPS membrane and recycled HIPS-R were 49% and 42%, respectively.

Recycled polystyrenes collected from the filling of packaging were dissolved in DMF to prepare flat sheet UF membranes through phase inversion using a film applicator to treat organic micropollutant-containing river water [155]. The polystyrene membrane fabricated with 16 wt% of polymer exhibited promising removal efficiency of ~40% for phenolic compounds. The colour removal of ~75% was comparable to that achieved by the membranes fabricated from commercial PES polymer and commercial CA membranes. The incorporation of hydroxyl-functionalized, single-walled carbon nanotubes (SWCNTs) within the matrix of 16 wt% polystyrene membrane further improved the retention of micropollutants. It was observed that the introduction of SWCNTs has negligible effects on the surface properties of the membrane, but the adsorptive capability of the nanomaterials improved the retention of microcontaminants through adsorption mechanism. 

Using phenol as a solvent and PEG as an additive, waste PET bottles were employed as a raw material for the fabrication of PET UF membrane through non-solvent-induced phase separation (NIPS) [156]. PEG acted as a plasticiser to reduce the stiffness besides improving the hydrophilicity of the PET membrane. The membrane properties were fine-tuned by altering the dope composition and additive concentration. It was observed that the pore size and water permeability of the PET membrane were enhanced by using non-solvent of lower polarity and PEG with higher molecular weight and at higher concentration. PET-based membrane derived from phenol-dissolved waste PET bottles was also prepared through thermally induced phase separation (TIPS), with cellulose acetate as a sub-polymer to improve mechanical strength and rice husk-derived silica as a pore-forming agent [157]. The addition of 1 wt% silica improved the surface hydrophilicity and the distribution of pores on the membrane surface, hence resulting in two-fold increment in its water permeability compared to that without silica additive.

**Figure 7:**
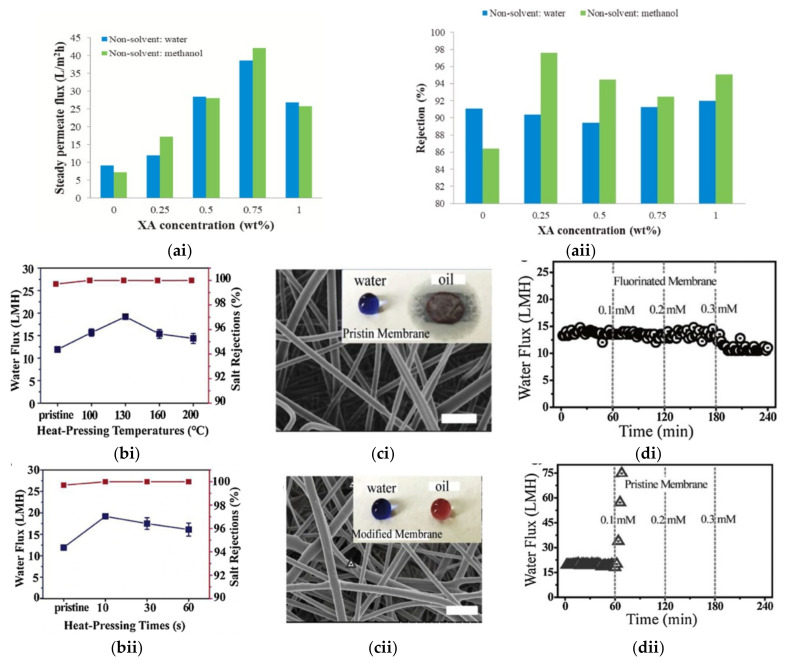
(**a**) (i) Steady permeate flux and (ii) diltiazem rejection of recycled PET NF membrane prepared from different concentration of XA and types of coagulants [158]. (**b**) Water flux and salt rejection of PET nanofibrous NF membrane as a function of (i) hot-pressing temperatures with duration of 10 s and (ii) hot-pressing duration at 130 °C (**c**) anti-wetting ability of (i) pristine and (ii) fluorinated membranes to water and oil; (**d**) water flux of (i) pristine and (ii) fluorinated membranes as function of surfactant concentration [160].

Kiani et al. first attempted the fabrication of NF membrane using post-consumer PET as a raw material and different concentration of xantham gum (XA) as a hydrophilic additive [158]. The presence of XA altered the membrane morphology and porosity, as it establishes hydrogen bonds with water molecule, thus increasing the exchange rate of solvent and non-solvent to promote large pore formation. However, beyond the threshold of 0.75 wt% XA, the increased solution viscosity slowed down the solvent and non-solvent exchange rate and suppressed macropore formation. Compared to water as non-solvent, coagulation using methanol offered XA-containing membranes with higher surface hydrophilicity, as part of the XA dissolved and leached into water during phase inversion. As shown in Figure 7ai, the recycled PET membrane with 0.75 wt% XA and coagulated in methanol achieved the highest permeate flux owing to its highest porosity and surface hydrophilicity. In term of the diltiazem rejection, rejections in the range of 89.4–92% was achieved by the neat recycled-PET membranes and 92.5–97.6% by the XA-added recycled PET membrane. Particularly, the membrane prepared with 0.25 wt% of XA using methanol as non-solvent exhibited the highest rejection of diltiazem (Figure 7aii, which is comparable to that achieved by the typical thin film composite or nanocomposite membranes prepared from commercial raw materials [159].

Nanofibrous membranes used for desalination through direct-contact membrane distillation were spun using PET recycled from soft drink bottles [161]. Post-treatment through hot pressing at 100–160 °C resulted in significant increase of water liquid entry pressure and mechanical strength. The post-treated PET nanofibrous membranes exhibited salt rejection of 99.9% and stable permeation of 11−23 L m^−2^ h^−1^ for long-term operation (Figure 7b). Particularly, hot pressing at 130 °C for 10 s induced moderate change of the membrane thickness, pore size, and porosity, hence achieving the optimal flux of 19.19 L m^−2^ h^−1^. 1H, 1H, 2H, 2H-perfluorodecyltriethoxysilane (FAS) was subsequently used for the membrane surface modification to render oleophobicity as the prerequisite to improve the wetting resistance of the fluorinated membrane against oil. As shown in Figure 7c, FAS-modified nanofibrous membrane exhibited high wetting resistance for water containing 0.3 M of sodium dodecyl sulphate (SDS) surfactant. The sharp increase in the water flux of pristine membrane shown in Figure 7d indicated the susceptibility of the membrane towards severe wetting even at low surfactant concentration. 

**Figure 8:**
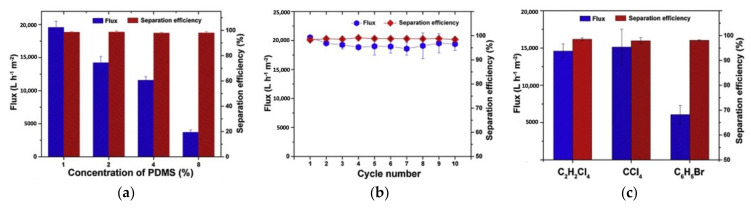
Flux and separation efficiency of PDMS-coated recycled PET nanofibrous membrane as a function of (**a**) PDMS concentration, (**b**) cycle number, and (**c**) types of hydrocarbon substances in oil–water separation [161].

In a similar study [161], nanofibrous membranes were fabricated from recycled PET, followed by dip-coating with polydimethylsiloxane (PDMS). The as-spun recycled PET nanofibre membranes possessed a low degree of interconnected network structure due to the incomplete elimination of trifluoroacetic acid (TFA) and dichloromethane (DCM) mixed solvent during electrospinning. Despite its high under-oil super-hydrophobicity, the as-spun recycled PET membrane was susceptible to oil adhesion. By controlling the amount of PDMS, the mechanical strength was improved owing to the strengthened interconnected bonding, and the anti-wetting properties were improved due to the greater membrane super-oleophilicity and under-oil super-hydrophobicity to facilitate fast oil permeation through the membrane. As shown in Figure 8a,b), with 1 wt% of PDMS coated on the recycled PET membrane, the oil–water separation was achieved with a flux and separation efficiency of ∼20,000 L m^−2^ h^−1^ and >98%, respectively, for up to ten cycles. However, the flux of bromobenzene–water separation became almost half of ethylene tetrachloride and carbon tetrachloride–water separation due to swelling effect of bromobenzene imposed on the PDMS layer, which reduced in turn the membrane pore size (Figure 8c).

Solvent-resistant UF membranes derived from post-consumer PET were prepared by NIPS technique using PEG and the mixture of trifluoroacetic acid (TFA)/dichloromethane (DCM) and organic compound as pore former, solvent, and non-solvent, respectively [162]. Reprecipitation of the PET obtained from waste plastic bottles was performed to obtain pure PET with yield of 99.5%, as evident from the Fourier-transformed infrared and thermogravimetric analyses. The crystallinity of PET decreased upon its conversion from bottle to membrane, indicating that the flexibility of the material was improved during the phase-inversion process. It was observed that with the same PET concentration of 10 wt%, the membrane precipitated in methanol non-solvent had larger pore size, hence achieving higher ethanol permeability compared to the membrane precipitated in ethanol. With pore size range of 35−40 nm, the methanol-precipitated PET membrane achieved ethanol permeability of 411 L m^−2^ h^−1^ bar^−1^, which was a more than four-fold increment compared to that of ethanol-precipitated PET membrane. When tested for the rejection of PEG from DMF solution, the membranes experienced an irreversible compaction of ~10% when the pressure was increased from 1 to 10 bar. At higher temperature of 140 °C, the DMF permeability was increased is due to the decrease in solution viscosity and occurrence of membrane swelling. However, the rejection was compromised due to the unfavourable change in its pore size as a result of membrane softening at high temperature.

**Figure 9:**
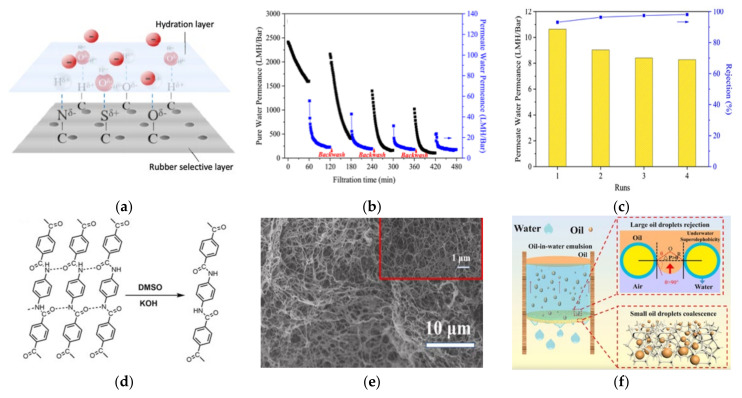
(**a**) Schematic illustration of hydration layer formation on rubber selective layer supported on ceramic substrate, (**b**) pure-water and permeate permeability of recycled rubber-derived NF membrane as a function of time, (**c**) permeate permeability of the membrane as a function of filtration cycles [163], (**d**) schematic illustration of the exfoliation of Kevlar fibre, (**e**) scanning electron microscope images of aramid nanofibre aerogel membrane, and (**f**) illustration of water-in-oil emulsion separation phenomena using aramid nanofibre aerogel membrane [164].

Recycled tyre-derived NF hollow fibre composite membrane was developed by Lin et al. using devulcanized rubber and rubber powder crushed from waste tyres as the rubber sources [163]. Upon dissolution by toluene, the rubber selective layer was formed on the internal surface of ceramic substrate, which acted as support to improve the mechanical strength. Via the capillary phenomenon, the rubber casting solution was drawn into the porous substrate, allowing the adhesion of rubber onto the substrate. It was observed that the thermal and solubility behaviours of the rubber were largely determined by the compositions of the waste rubber. Compared the devulcanized recycled rubber, the solubility of rubber powder was inferior due to its crosslinked 3-D structure. In the filtration using MB dye solution, the dye rejection improved linearly along with the permeability reduction as the number of internal coating increased due to the dense rubber selective layer. On the other hand, when the rubber concentration was increased from 6 wt% to 12 wt%, the pure-water permeability increased from 470.2 to 1596.1 L m^−2^ h^−1^ bar^−1^, and the permeability of the dye solution increased from 3.9 to 10.6 L h^−1^ m^−2^bar^−1^ owing to the increase in the surface hydrophilic groups, which form the hydration layer, as schematically illustrated in Figure 9a. The antifouling properties rendered by the hydration layer allowed permeance recovery, reaching over 80% upon washing. The hydration layer also acts as barrier to alleviate the adsorption of MB molecules on the membrane surface Figure 9b. The unvulcanised rubber-derived NF membrane with 12 wt% rubber concentration and three times of internal coating achieved MB rejection of 93.1%. As shown in Figure 9c, after three cycles of filtration, the membrane permeability could be maintained at 8.3 L m^−2^ h^−1^ bar^−1^ with a MB rejection 98.1%.

A three-dimensional porous aerogel membrane derived from waste Kevlar fibre was reported for surfactant stabilized oil-in-water emulsions separation [164]. The hydrogel was obtained by dissolving the aramid fibre in the mixture of potassium hydroxide (KOH)/DMSO to allow exfoliation of the fibre to form nanosized fibre, as depicted in Figure 9d, followed by solvent exchange to form crosslinked networks. The aerogel membrane was formed from the coating and freeze-drying of the aramid nanofibre aerogel. The resultant sponge-like porous aerogel membrane with interconnected nanofibres (Figure 9e) was in a micropore size range and characterized by super light-weight, high-porosity, and high superhydrophilic/underwater superoleophobic property. The aramid fibre also rendered the membrane with high thermal stability. The aerogel membrane overcame the limitation of molecular-sieving membranes by offering tortuously porous structures to facilitate tiny droplets separation via coalescence in the tortuous channels. The separation efficiency of >98.0% was maintained after 10 filtration cycles. As illustrated in Figure 9f, upon the contact of water in the oil-in-water emulsion with the aerogel membrane, a steady water/solid composite interface was formed, thus hampering the passage of the large oil droplet. On the other hand, the tiny emulsion droplets that penetrated across the membrane collided and adhered to each other in tortuous microchannels of the aerogel membrane, forming larger droplets that can coalesce in the depth of the aerogel membrane. An efficient emulsion separation of 98.1% with flux of 1940 L m^−2^ h^−1^ was achieved by virtue of its promising surface and structural attributes.

## 4. Challenges and Future Directions

The advantages of waste reutilization have extensive reach. It is not only able to mitigate worsening problems from unsustainable and improper waste management but also can help a business in gaining economic benefits in the long run while achieving a cleaner production through a sustainable chain. Waste recycling and upcycling offer a platform to promote commercial value, especially for agricultural wastes that have been known to have little or no commercial value. For instance, the conversion of cotton microdust to cellulose serves as a tool to improve the economics of cotton mills. On the other hand, most plastic wastes, such as used PET bottles, have been recycled into lower-grade products; hence, the profit involved in the recycled material production is relatively low. The conversion of waste to high-grade product deserves greater attention and should be further promoted. The value-added products derived from these wastes hold greater potentials to offset the cost of recycling. The transformation from waste down-cycling to form low-value product to the production of high-value membrane can also serve as a driving force to provide a strong economic incentive to improve the recycling rate. As summarized in Table 1, encouraging progresses have been made in the development of waste-derived polymeric membranes based on keratin, cellulose, plastics, and polymers. A better understanding of the nature of waste substances, the complex functional structures, and their extraction methods would allow their expanded applications. Some challenges and limitations with the corresponding suggestions for improvement and future way forward have been identified to help in promoting waste reutilization in the membrane industry.

The polymer industry has experienced exponential growth in the last 50 years, with synthetic and engineering materials, such as PVDF and PSF, taking the lead in membrane separation applications. The main motivation of utilizing recycled materials in membrane manufacturing is the reduction in the usage of commercial feedstock. Membrane manufacturers play critical role in seeing the benefits that they can harness from the waste resources. Since the priority is to move from lab-scale exploration to a realistic industry-scale production, the assessment of these sustainable strategies must not only consider the small-scale and idealized performance of the membranes, but the innovation must also be translated into a technically feasible membrane manufacturing process. It is worth it for the relevant stakeholders to intensify the research in waste recycling to gain a deeper knowledge in this area and narrow the circularity gap. For the related industries involved in the membrane supply chain that are interested in undertaking recycling activities in their production, waste recycling incentive in the form of tax exemption can be a useful initiative to speed up the implementation. The relevance of recycling incentive scheme in stimulating the initiation and adoption of solid waste in membrane production industries as well as the sustainability of the efforts after the withdrawal of incentive scheme should be studied. 

Solid waste resources are diverse and characterized by drastically different properties. Through the identification of an appropriate extraction or dissolution method, the desired polymeric components can be obtained and used as alternative of commercial polymer raw materials. Nevertheless, the decision on the type of polymers to target is not only based on its suitability, prices, and the relevant technological setup, but it is also affected by the composition and purity of the input materials. It is known that the impurities present in some waste products could impose significant effects on the physical properties of the membrane. For example, the presence of lignin globules in some of the waste cellulose source can result in high film-surface roughness, but the mechanical properties of the resultant film can also be improved with the presence of trace amounts of lignin [165]. Some of the additives introduced during the recycling process, such as reinforcing fillers, plasticisers, and pigments used for plastic recycling, may act as suppressors of macrovoids or pore-forming agents [166]. Consequently, the membrane morphology and texture properties can be quite different from those fabricated from commercial raw material. Purification of waste may be required in some cases to achieve better homogeneity and to get rid of their undesirable effect on the resultant membrane. As the structural properties of membranes are closely related to their separation performances, it is advisable to perform a thorough compositional analysis to obtain sufficient details for a better correlation with the membrane performances.

The success of waste recycling and reutilization strategy in the context of membrane production is intimately dependant on the formability of the membranes from these sources. The construction of ternary phase diagram can provide more fundamental understanding on the interaction of the recycled raw materials with the solvent and non-solvent components in the dope solutions during phase inversion. While plastic waste can be generally prepared using the same approach as for other commercial thermoplastic polymers, the low molecular weight of most biopolymers has made the membrane fabrication process not as scalable as that of using commercial materials. For example, the fabrication of nanofibrous membranes using keratin with molecular weight in the range of 10–60 kDa is difficult. In most preparations, the blending of suitable commercial polymers that can dissolve in a common solvent is required. The scope of study related to dope formulation and membrane formability certainly deserves better attention, but currently, it has not been sufficiently covered in most studies. Furthermore, due to the undisclosed membrane composition and formulations, it is difficult to reach consensus among the membrane manufacturers on finding appropriate strategies to identify the most suitable source and amount of solid waste to be incorporated in the existing production.

Other than identifying the right material and verifying their properties, the preparation methods should be less complex and require less sophisticated instrumentation so that they can be easily reproduced and scaled up. Priority should be given to the waste that requires minimum and less tedious pretreatment or processing. The dissolution of waste in their original form remains challenges due to their tough structure and recalcitrant nature to various solvents and treatment conditions. The recovery of some waste for the attainment of high-quality raw material for membrane fabrication is a notoriously difficult. Biopolymers, such as cellulose, which normally is extracted from lignocellulosic biomass, can only be obtained after going through a series of intensive and wet chemistry-based treatment and sophisticated routes where a huge amount of reagents are required for the delignification and extraction processes. Many of the solvents used, such as toluene, benzene, and chlorite, have been related to the production of unwanted byproducts. The strong foundation for the utilization waste-derived materials as raw material of high-value products is associated to the abundance and availability of these wastes. This economic advantage should not be offset by the cost involved in the volarisation of the waste. It is therefore important to look into the development of more effective and eco-friendly techniques of waste processing.

In line with the call of sustainable membrane production using the green chemistry principle [167], more efforts should also be directed to the establishment of less energy intensive waste processes using less toxic solvent at low quantity and low concentration. Polymeric membrane manufacturing is still generally far from sustainable and environmentally friendly [168]. Particularly, most of the commercially used solvents are toxic and strictly regulated in their industrial use. While the reutilization of bio-based waste as polymeric raw material is expected to bring down the unfavourable effects of engineering polymers to certain extent, the use of green solvent in the dissolution of solid waste-derived raw material during membrane fabrication is also imperative to maximize the process sustainability. A number of studies have demonstrated the feasibility of green solvent or the less toxic solvent in the preparation of polymer dope solution [169]. It is worth noting that, although IL has been well recognized as a versatile green solvent due to its high dissolution capacity and recyclability, the current production cost of ionic liquid is still the major hindrance for its commercial viability. It is expected that the application of IL will only become more common if a fast and efficient extraction method can be scaled up for IL recovery. Molten inorganic salt is another cost-effective and environmentally friendly alternative, as the high aqueous solubility of molten salts facilitates easy removal and efficient isolation from the product [170]. A salient point to be highlighted is that the waste solubilisation and extraction reported in literature is based only on a small quantity at the laboratory level. In the same vein, while the reutilization of waste for membrane fabrication is touted as a sustainable approach to curb environmental issues, its actual effect in increasing sustainability of membrane production has not been quantitatively assessed at a practical scale due to the lack of industrial studies and data. Life cycle assessment (LCA) approach, which looks into multiple interacting parts, may serve as a basic tool to study and evaluate the environmental impacts of the manufacturing processes and the final product.

Another key point to increase the adoption of a new membrane material into the existing manufacturing process is the adaptability of the materials to the already-established membrane fabrication technique. It has been generally observed that electrospinning has been widely explored, in addition to the typically used phase-inversion technique, for the fabrication of waste-derived polymeric membranes. Owing to its versatility in handling a wide range of polymer dope and flexibility in fine tuning membrane properties through the optimization of its operating conditions, electrospinning has received increasing attention in polymeric membrane fabrication. However, compared to the mature, non-solvent-induced, and thermally induced precipitation technique, electrospinning fabrication technique has not earned a significant position in today’s liquid separation membrane market. Looking on the bright side, increasing efforts have also been made in tuning the fabrication method to produce membranes with desired, tailor-made properties. This offers a great opportunity to cater to the properties of the polymeric materials obtained from waste resources. Three pillars support the enabling of this strategy, i.e., waste collection, treatment, and conversion to product. For membrane manufacturing, the pillars can be summarized in more specific processes, i.e., obtaining and transportation of waste materials, pre-treatment and initial separation to obtain the desired raw material, and finally the production stage, where the raw materials are fabricated into membrane products, which are used in a new chain of application in other industries. Waste collection, storage, and transport must be properly integrated into this system, as they are essential elements in ensuring the sustainable source for production. As it may be a significant loss of target materials occurred at recycling stages, it is imperative to ensure the efficient handling of wastes of various sources during this stage.

While the current state-of-the-art technologies offer many possibilities for the application of various waste for the development of high-value products, the scientific findings of research and technological development offer opportunities to consolidate these technologies on industrial environment. Comparisons among studies are essential to benchmark the performances of the similar materials. However, most presented comparisons served only as nominal, as the preparation conditions, including the solvent system used for the dissolutions of the waste materials, membrane fabrication parameters, and performance measurement parameters, varied from one study to another. Nevertheless, the reliability of the physical properties and separation performances of the waste-derived membranes can be enhanced to a greater extent if side-by-side comparisons are made with the membranes produced from the same but commercially available material. There have been some debates about the reliability of most post-consumer and post-industry recycled materials as membrane feedstock, particularly their applicability for long-term operation. It has been pointed out that recycled materials generally have deteriorated mechanical strength and resistance to oxidation compared to their virgin counterpart, hence lower strength rating and life prediction. A comprehensive comparison can eliminate these uncertainties and further boost the confidence of industries and stakeholder in adopting the idea.

Efforts have been invested in searching for waste materials in which polymeric feedstock can be potentially recovered, recycled, or extracted. However, it is generally observed that the development of waste-derived membranes receives significantly low attention in relation to the development of new membrane materials and enhanced manufacturing techniques that have the potential to overcome the trade-off limit or address other inherent membrane issues, such as fouling. The overall literature search also revealed that waste-derived membranes have rarely been explored in the fabrication of thin film composite (TFC) membrane for RO and forward osmosis or hydrophobic microporous membrane for membrane distillation. From the TFC RO membrane recycling point of view, most studies have been focused on the recycling of end-of-life RO membranes, where the waste membranes are transformed into NF, UF, or MF membranes for either lower grade treatment or for new applications. In fact, waste reutilization for RO membrane fabrication can also generate economic interest. It holds a great potential to utilize the waste-derived electrospun polymeric membranes as the substrate for thin film composite membrane. On the other hand, attempts can be ventured to evaluate the potential of plastic waste that are normally hydrophobic in nature for the fabrication of microporous membranes used for membrane distillation. Some relatively uncommon and novel membranes, such as waste-derived aerogel membranes, have also been reported. However, despite the unique structural and textural properties, aerogel membrane has been rarely reported from liquid separation, primarily due to the challenges in its fabrication, such as complication in preparation and difficulty in controlling the desired structure.

It should be highlighted that waste recycling and reutilization for commercial product manufacturing involves a multidisciplinary perspective. It is not only related to the technological elements but also requires the readiness of the community, especially of those who are involved in post-consumer waste centralization and collection, stakeholders, and industries. To date, although valuable research progresses based on a wide range of solid waste have been increasingly reported for their usefulness as raw material for polymeric membrane fabrication, the research and development in this area have so far been focused on providing illustrative evidences through the demonstration of the preparation techniques, including membrane formulation and solvent selection, as well as comparisons between waste-derived and commercial material-derived polymeric membrane in terms of their properties and separation performances. Generally, besides the technical challenges, efforts are still lacking in evaluating the economic feasibility of this approach. This gap suggests a need for a framework not only to assess their contributions to the circular economy but also to look into the potential of reaping the practical cost benefits and readiness for implementation at the manufacturer level. Such framework will ensure a more successful promotion on a larger scale in the longer term. Adequate economic and thermodynamic evaluations should also be performed on processes of converting the waste into the corresponding polymeric feedstock.

## 5. Conclusions

With the increasing driving force for membrane-based separation processes, the availability of waste-derived natural resources can offer pragmatic motivations for sustainable membrane development. As sustainability becomes the watchword of current research and development in almost all fields, various solid waste generated from numerous industries can be regarded as precious commodities and innovative resources. Waste reutilization in membrane fabrication can be closely associated to United Nation’s Sustainable Development Goals (SDGs) 12, which aims to ensure sustainable consumption and production patterns. The reutilization of waste not only could protect the environment but also conserve resources. By reutilizing waste from various sources for the production of high-value polymeric membranes with tremendous market potential on the global scale, the demand of raw materials in the fabrication process can be reduced, and the impact of environmental pollution due to the solid waste disposal can also be minimized. Although membrane fabrication techniques have been well established, there are still many challenges and bottlenecks to be tackled. It is important to look at the perspective of these wastes as raw materials for membrane by the academic sector on one hand and the interest of the membrane manufacturing industry on the other hand, as the decision of industries and manufacturers is another critical factor in dictating the success of its implementation. It is difficult to observe radical changes in the current practice within the coming five years, but with the mutual interest of all parties, it is expected to witness some positive and steady progress and acceptance of waste reutilization in membrane industries in capturing the real benefits of this effort.

## Figures and Tables

**Table 1:** Summary of polymeric membranes prepared from waste sources of keratin, cellulose, polymer, and plastic.

Materials	Solvent	Water Source	Membrane	Removal/Separation	Performance Indicator	Refs.
Keratin	IL	Dyed wool fabric	NF nanofibre membrane	-	Bacteria inhibition rate: *E. coli*: 89.2% *S. aureus*: 60.7	[137]
Keratin	Formic acid	Goat hair	Polyamide/keratin electro spun membrane	Tannery dye removal	Rejection: 100%	[138]
Cellulose	NMP	OPEFB	UF	Dye removal	MB removal: 34.9%	[140]
Cellulose	-	Kapok fibre	Self-assembled CNC membrane	Dye removal	MB removal: 85%	[141]
Cellulose	DMSO	Non-dye cotton bed sheet	UF	-	PEG 35 kD rejection: ~90%	[142]
Cellulose	NaOH/urea	Recycled newspaper	UF	-	PWP: 0.35 L m^−2^ h^−1^ bar^−1^	[148]
Cellulose	NaOH/urea	Recycled newspaper	Photocatalytic membrane, TiO_2_ nanorod additive	Phenol removal	Phenol degradation: 96.6% (UV) 78.8% (Visible light) PWF: 4.12 L m^−2^ h^−1^	[144]
Cellulose	DMF	Waste cigarette filter	Electrospun stainless steel supported membrane	Oil/water emulsion separation	Separation efficiency: >99% Flux: > 100 L m^−2^ h^−1^	[149]
Cellulose	DMF	Waste cigarette filter	UF	Oil/water emulsion separation	Separation efficiency: >94% P: > 180 L m^−2^ h^−1^ bar^−1^	[150]
PVC	NMP	Campus post-consumer disposal	UF, GA additive	Humic acid removal	HA rejection: 96% Flux: 98 L m^−2^ h^−1^	[153]
PET	Phenol	Post-consumer bottle	UF, PEG4000 additive	BSA removal	BSA rejection: 90%	[158]
HIPS	DMF	Recycled	UF	Humic acid removal	*R_T_*= 49% HA rejection: 96%	[128]
PS	DMF	Packaging filling	UF	Micropollutant-containing river wastewater treatment	Phenolic compound rejection: ~40% Colour rejection: ~70%	[157]
PET	TFA	Post-consumer bottle	NF, XA additive	Diltiazem-containing solution	Diltiazem rejection:	[156]
PET	TFA	Post-consumer soft drink bottle	Electrospun NF, hot pressed and fluorinated	Desalination	Permeation: 11−23 L m^−2^ h^−1^ Salt rejection: >99.9%	[161]
PET	TFA and dichloromethane (DCM)	Post-consumer bottle	Electrospun NF, PDMS coating	Oil/water separation	Flux: 20,000 L m^−2^ h^−1^ Separation efficiency: >98%	[162]
Rubber	Toluene	Recycled, unvulcanised tyre	NF	MB removal	PWP: 1596.1 L m^−2^ h^−1^ bar^−1^ Permeability: 10.6 L m^−2^ h^−1^ bar^−1^ MB rejection: 93%	[163]
Aramid fibre	KOH/DMSO	Kevlar	Aerogel membrane	Oil/water emulsion separation	Separation efficiency: >98% Flux: > 1940 L m^−2^ h^−1^	[164]

## Data Availability

Not applicable.

## References

[1] Abu-Bakar H., Williams L., Hallett S.H. (2021). A review of household water demand management and consumption measurement. J. Clean. Prod..

[2] Muhammad B. (2019). Energy consumption, CO2 emissions and economic growth in developed, emerging and Middle East and North Africa countries. Energy.

[3] Mathai M.V., Isenhour C., Stevis D., Vergragt P., Bengtsson M., Lorek S., Mortensen L.F., Coscieme L., Scott D., Waheed A. (2021). The Political Economy of (Un)Sustainable Production and Consumption: A Multidisciplinary Synthesis for Research and Action. Resour. Conserv. Recycl..

[4] Sharma S., Basu S., Shetti N.P., Kamali M., Walvekar P., Aminabhavi T.M. (2020). Waste-to-energy nexus: A sustainable development. Environ. Pollut..

[5] Rautela R., Arya S., Vishwakarma S., Lee J., Kim K.H., Kumar S. (2021). E-waste management and its effects on the environment and human health. Sci. Total Environ..

[6] Akan O.D., Udofia G.E., Okeke E.S., Mgbechidinma C.L., Okoye C.O., Zoclanclounon Y.A.B., Atakpa E.O., Adebanjo O.O. (2021). Plastic waste: Status, degradation and microbial management options for Africa. J. Environ. Manag..

[7] Alshehrei F., Ameen F. (2021). Vermicomposting: A management tool to mitigate solid waste. Saudi J. Biol. Sci..

[8] Zhang J., Qin Q., Li G., Tseng C.H. (2021). Sustainable municipal waste management strategies through life cycle assessment method: A review. J. Environ. Manag..

[9] Sharma H.B., Vanapalli K.R., Samal B., Cheela V.R.S., Dubey B.K., Bhattacharya J. (2021). Circular economy approach in solid waste management system to achieve UN-SDGs: Solutions for post-COVID recovery. Sci. Total Environ..

[10] Luttenberger L.R. (2020). Waste management challenges in transition to circular economy—Case of Croatia. J. Clean. Prod..

[11] Agyabeng-Mensah Y., Tang L., Afum E., Baah C., Dacosta E. (2021). Organisational identity and circular economy: Are inter and intra organisational learning, lean management and zero waste practices worth pursuing?. Sustain. Prod. Consum..

[12] Jõgi K., Bhat R. (2020). Valorization of food processing wastes and by-products for bioplastic production. Sustain. Chem. Pharm..

[13] Goh P.S., Ismail A.F. (2017). A review on inorganic membranes for desalination and wastewater treatment. Desalination.

[14] Goh P.S., Ismail A.F., Ng B.C., Abdullah M.S. (2019). Recent progresses of forward osmosis membranes formulation and design for wastewater treatment. Water.

[15] Goswami K.P., Pugazhenthi G. (2020). Credibility of polymeric and ceramic membrane filtration in the removal of bacteria and virus from water: A review. J. Environ. Manag..

[16] Nunes S.P., Culfaz-Emecen P.Z., Ramon G.Z., Visser T., Koops G.H., Jin W., Ulbricht M. (2020). Thinking the future of membranes: Perspectives for advanced and new membrane materials and manufacturing processes. J. Membr. Sci..

[17] Kugarajah V., Ojha A.K., Ranjan S., Dasgupta N., Ganesapillai M., Dharmalingam S., Elmoll A., Hosseini S.A., Muthulakshmi L., Vijayakumar S. (2021). Future applications of electrospun nanofibers in pressure driven water treatment: A brief review and research update. J. Environ. Chem. Eng..

[18] Sarbatly R., Sariau J., Alam M.F.I. (2021). Advances in nanofiber membrane. Mater. Today Proc..

[19] Bandehali S., Sanaeepur H., Ebadi Amooghin A., Shirazian S., Ramakrishna S. (2021). Biodegradable polymers for membrane separation. Sep. Purif. Technol..

[20] Fredi G., Dorigato A. (2021). Recycling of bioplastic waste: A review. Adv. Ind. Eng. Polym. Res..

[21] Udayakumar G.P., Muthusamy S., Selvaganesh B., Sivarajasekar N., Rambabu K., Sivamani S., Sivakumar N., Maran J.P., Hosseini-Bandegharaei A. (2021). Ecofriendly biopolymers and composites: Preparation and their applications in water-treatment. Biotechnol. Adv..

[22] Patel S.H., Xanthos M. (2001). Environmental Issues in Polymer Processing: A Review on Volatile Emissions and Material/Energy Recovery Options.

[23] Mansoori S., Davarnejad R., Matsuura T., Ismail A.F. (2020). Membranes based on non-synthetic (natural) polymers for wastewater treatment. Polym. Test..

[24] Debnath B., Haldar D., Purkait M.K. (2021). A critical review on the techniques used for the synthesis and applications of crystalline cellulose derived from agricultural wastes and forest residues. Carbohydr. Polym..

[25] Bushra R., Mohamad S., Alias Y., Jin Y., Ahmad M. (2021). Current approaches and methodologies to explore the perceptive adsorption mechanism of dyes on low-cost agricultural waste: A review. Microporous Mesoporous Mater..

[26] Freitas L.C., Barbosa J.R., da Costa A.L.C., Bezerra F.W.F., Pinto R.H.H., de Carvalho Junior R.N. (2021). From waste to sustainable industry: How can agro-industrial wastes help in the development of new products?. Resour. Conserv. Recycl..

[27] Kwikima M.M., Mateso S., Chebude Y. (2021). Potentials of Agricultural wastes as the ultimate alternative adsorbent for Cadmium removal from wastewater. A review. Sci. Afr..

[28] Lewoyehu M. (2021). Comprehensive Review on Synthesis and Application of Activated Carbon from Agricultural Residues for the Remediation of Venomous Pollutants in Wastewater. J. Anal. Appl. Pyrolysis.

[29] Solangi N.H., Kumar J., Mazari S.A., Ahmed S., Fatima N., Mubarak N.M. (2021). Development of fruit waste derived bio-adsorbents for wastewater treatment: A review. J. Hazard. Mater..

[30] Kadhom M., Albayati N., Alalwan H., Al-Furaiji M. (2020). Removal of dyes by agricultural waste. Sustain. Chem. Pharm..

[31] Zhang K., Zhang F., Wu Y.R. (2021). Emerging technologies for conversion of sustainable algal biomass into value-added products: A state-of-the-art review. Sci. Total Environ..

[32] Yan G., Chen B., Zeng X., Sun Y., Tang X., Lin L. (2020). Recent advances on sustainable cellulosic materials for pharmaceutical carrier applications. Carbohydr. Polym..

[33] Abdulyekeen K.A., Umar A.A., Patah M.F.A., Daud W.M.A.W. (2021). Torrefaction of biomass: Production of enhanced solid biofuel from municipal solid waste and other types of biomass. Renew. Sustain. Energy Rev..

[34] Sharma P., Gaur V.K., Sirohi R., Varjani S., Hyoun Kim S., Wong J.W.C. (2021). Sustainable processing of food waste for production of bio-based products for circular bioeconomy. Bioresour. Technol..

[35] Jannat N., Hussien A., Abdullah B., Cotgrave A. (2020). Application of agro and non-agro waste materials for unfired earth blocks construction: A review. Constr. Build. Mater..

[36] Al-Fakih A., Mohammed B.S., Liew M.S., Nikbakht E. (2019). Incorporation of waste materials in the manufacture of masonry bricks: An update review. J. Build. Eng..

[37] Li L., Zuo J., Duan X., Wang S., Hu K., Chang R. (2021). Impacts and mitigation measures of plastic waste: A critical review. Environ. Impact Assess. Rev..

[38] Sharma B., Shekhar S., Sharma S., Jain P. (2021). The paradigm in conversion of plastic waste into value added materials. Clean. Eng. Technol..

[39] Al Rayaan M.B. (2021). Recent advancements of thermochemical conversion of plastic waste to biofuel-A review. Clean. Eng. Technol..

[40] Zulkernain N.H., Gani P., Chuck Chuan N., Uvarajan T. (2021). Utilisation of plastic waste as aggregate in construction materials: A review. Constr. Build. Mater..

[41] Wang J., Qian W., He Y., Xiong Y., Song P., Wang R.M. (2017). Reutilization of discarded biomass for preparing functional polymer materials. Waste Manag..

[42] Maiti A., Pandey A. (2021). Polymer and Waste Plastic in Membranes. Reference Module in Materials Science and Materials Engineering.

[43] Donato R.K., Mija A. (2020). Keratin associations with synthetic, biosynthetic and natural polymers: An extensive review. Polymers.

[44] Reddy C.C., Khilji I.A., Gupta A., Bhuyar P., Mahmood S., AL-Japairai K.A.S., Chua G.K. (2021). Valorization of keratin waste biomass and its potential applications. J. Water Process Eng..

[45] Shavandi A., Silva T.H., Bekhit A.A., Bekhit A.E.D.A. (2017). Keratin: Dissolution, extraction and biomedical application. Biomater. Sci..

[46] Donner M.W., Arshad M., Ullah A., Siddique T. (2019). Unravelled keratin-derived biopolymers as novel biosorbents for the simultaneous removal of multiple trace metals from industrial wastewater. Sci. Total Environ..

[47] Gaidau C., Epure D.G., Enascuta C.E., Carsote C., Sendrea C., Proietti N., Chen W., Gu H. (2019). Wool keratin total solubilisation for recovery and reintegration—An ecological approach. J. Clean. Prod..

[48] Ma B., Qiao X., Hou X., Yang Y. (2016). Pure keratin membrane and fibres from chicken feather. Int. J. Biol. Macromol..

[49] Holkar C.R., Jain S.S., Jadhav A.J., Pinjari D.V. (2018). Valorization of keratin based waste. Process Saf. Environ. Prot..

[50] Chojnacka K., Skrzypczak D., Mikula K., Witek-Krowiak A., Izydorczyk G., Kuligowski K., Bandrów P., Kułażyński M. (2021). Progress in sustainable technologies of leather wastes valorization as solutions for the circular economy. J. Clean. Prod..

[51] Aluigi A., Sotgiu G., Torreggiani A., Guerrini A., Orlandi V.T., Corticelli F., Varchi G. (2015). Methylene Blue Doped Films of Wool Keratin with Antimicrobial Photodynamic Activity. ACS Appl. Mater. Interfaces.

[52] Balaji S., Kumar R., Sripriya R., Kakkar P., Ramesh D.V., Reddy P.N.K., Sehgal P.K. (2012). Preparation and comparative characterization of keratin-chitosan and keratin-gelatin composite scaffolds for tissue engineering applications. Mater. Sci. Eng. C.

[53] Park M., Shin H.K., Kim B.S., Kim M.J., Kim I.S., Park B.Y., Kim H.Y. (2015). Effect of discarded keratin-based biocomposite hydrogels on the wound healing process in vivo. Mater. Sci. Eng. C.

[54] Ramirez D.O.S., Carletto R.A., Tonetti C., Giachet F.T., Varesano A., Vineis C. (2017). Wool keratin film plasticized by citric acid for food packaging. Food Packag. Shelf Life.

[55] Feroz S., Muhammad N., Ranayake J., Dias G. (2020). Keratin—Based materials for biomedical applications. Bioact. Mater..

[56] Lazarus B.S., Chadha C., Velasco-Hogan A., Barbosa J.D.V., Jasiuk I., Meyers M.A. (2021). Engineering with keratin: A functional material and a source of bioinspiration. iScience.

[57] Rajabi M., Ali A., McConnell M., Cabral J. (2020). Keratinous materials: Structures and functions in biomedical applications. Mater. Sci. Eng. C.

[58] Sharma S., Gupta A., Chik S.M.S.T., Kee C.G., Mistry B.M., Kim D.H., Sharma G. (2017). Characterization of keratin microparticles from feather biomass with potent antioxidant and anticancer activities. Int. J. Biol. Macromol..

[59] Tran C.D., Mututuvari T.M. (2016). Cellulose, Chitosan and Keratin Composite Materials: Facile and Recyclable Synthesis, Conformation and Properties. ACS Sustain. Chem. Eng..

[60] Esparza Y., Bandara N., Ullah A., Wu J. (2018). Hydrogels from feather keratin show higher viscoelastic properties and cell proliferation than those from hair and wool keratins. Mater. Sci. Eng. C.

[61] Ozaki Y., Takagi Y., Mori H., Hara M. (2014). Porous hydrogel of wool keratin prepared by a novel method: An extraction with guanidine/2-mercaptoethanol solution followed by a dialysis. Mater. Sci. Eng. C.

[62] Ding S., Sun Y., Chen H., Xu C., Hu Y. (2019). An ultrasonic-ionic liquid process for the efficient acid catalyzed hydrolysis of feather keratin. Chin. J. Chem. Eng..

[63] Schindl A., Hagen M.L., Muzammal S., Gunasekera H.A.D., Croft A.K. (2019). Proteins in ionic liquids: Reactions, applications, and futures. Front. Chem..

[64] Idris A., Vijayaraghavan R., Rana U.A., Patti A.F., MacFarlane D.R. (2014). Dissolution and regeneration of wool keratin in ionic liquids. Green Chem..

[65] Jiang Z., Yuan J., Wang P., Fan X., Xu J., Wang Q., Zhang L. (2018). Dissolution and regeneration of wool keratin in the deep eutectic solvent of choline chloride-urea. Int. J. Biol. Macromol..

[66] Nuutinen E.M., Willberg-Keyriläinen P., Virtanen T., Mija A., Kuutti L., Lantto R., Jääskeläinen A.S. (2019). Green process to regenerate keratin from feathers with an aqueous deep eutectic solvent. RSC Adv..

[67] Xie H., Li S., Zhang S. (2005). Ionic liquids as novel solvents for the dissolution and blending of wool keratin fibres. Green Chem..

[68] Brown E.M., Pandya K., Taylor M.M., Liu C.-K. (2016). Comparison of Methods for Extraction of Keratin from Waste Wool. Agric. Sci..

[69] Shen B., Zhang D., Wei Y., Zhao Z., Ma X., Zhao X., Wang S., Yang W. (2019). Preparation of Ag doped keratin/PA6 nanofiber membrane with enhanced air filtration and antimicrobial properties. Polymers.

[70] Sinkiewicz I., Śliwińska A., Staroszczyk H., Kołodziejska I. (2017). Alternative Methods of Preparation of Soluble Keratin from Chicken Feathers. Waste Biomass Valorization.

[71] Fagbemi O.D., Sithole B., Tesfaye T. (2020). Optimization of keratin protein extraction from waste chicken feathers using hybrid pre-treatment techniques. Sustain. Chem. Pharm..

[72] Saha S., Zubair M., Khosa M.A., Song S., Ullah A. (2019). Keratin and Chitosan Biosorbents for Wastewater Treatment: A Review. J. Polym. Environ..

[73] Khosa M.A., Wu J., Ullah A. (2013). Chemical modification, characterization, and application of chicken feathers as novel biosorbents. RSC Adv..

[74] Hussain F.S., Memon N., Khatri Z., Memon S. (2020). Solid waste-derived biodegradable keratin sponges for removal of chromium: A circular approach for waste management in leather industry. Environ. Technol. Innov..

[75] Zahara I., Arshad M., Naeth M.A., Siddique T., Ullah A. (2021). Feather keratin derived sorbents for the treatment of wastewater produced during energy generation processes. Chemosphere.

[76] Aluigi A., Rombaldoni F., Tonetti C., Jannoke L. (2014). Study of Methylene Blue adsorption on keratin nanofibrous membranes. J. Hazard. Mater..

[77] Aluigi A., Varesano A., Vineis C., del Rio A. (2017). Electrospinning of immiscible systems: The wool keratin/polyamide-6 case study. Mater. Des..

[78] Ariffin H., Hassan M.A., Shah U.K.M., Abdullah N., Ghazali F.M., Shirai Y. (2008). Production of bacterial endoglucanase from pretreated oil palm empty fruit bunch by bacillus pumilus EB3. J. Biosci. Bioeng..

[79] Wang S., Dai G., Yang H., Luo Z. (2017). Lignocellulosic biomass pyrolysis mechanism: A state-of-the-art review. Prog. Energy Combust. Sci..

[80] Phromphithak S., Onsree T., Tippayawong N. (2021). Machine learning prediction of cellulose-rich materials from biomass pretreatment with ionic liquid solvents. Bioresour. Technol..

[81] Baghel R.S., Reddy C.R.K., Singh R.P. (2021). Seaweed-based cellulose: Applications, and future perspectives. Carbohydr. Polym..

[82] Fan J.S., Li Y.H. (2012). Maximizing the yield of nanocrystalline cellulose from cotton pulp fiber. Carbohydr. Polym..

[83] Srasri K., Thongroj M., Chaijiraaree P., Thiangtham S., Manuspiya H., Pisitsak P., Ummartyotin S. (2018). Recovery potential of cellulose fiber from newspaper waste: An approach on magnetic cellulose aerogel for dye adsorption material. Int. J. Biol. Macromol..

[84] Medronho B., Lindman B. (2015). Brief overview on cellulose dissolution/regeneration interactions and mechanisms. Adv. Colloid Interface Sci..

[85] Peng B., Yao Z., Wang X., Crombeen M., Sweeney D.G., Tam K.C. (2020). Cellulose-based materials in wastewater treatment of petroleum industry. Green Energy Environ..

[86] Homem N.C., Amorim M.T.P. (2019). Synthesis of cellulose acetate using as raw material textile wastes. Materials Today: Proceedings.

[87] Eduok U., Abdelrasoul A., Shoker A., Doan H. (2021). Recent developments, current challenges and future perspectives on cellulosic hemodialysis membranes for highly efficient clearance of uremic toxins. Mater. Today Commun..

[88] Silva M.A., Belmonte-Reche E., de Amorim M.T.P. (2021). Morphology and water flux of produced cellulose acetate membranes reinforced by the design of experiments (DOE). Carbohydr. Polym..

[89] Chevalier Q., el Hadri H., Petitjean P., Bouhnik-Le Coz M., Reynaud S., Grassl B., Gigault J. (2018). Nano-litter from cigarette butts: Environmental implications and urgent consideration. Chemosphere.

[90] Puls J., Wilson S.A., Hölter D. (2011). Degradation of Cellulose Acetate-Based Materials: A Review. J. Polym. Environ..

[91] Rebischung F., Chabot L., Biaudet H., Pandard P. (2018). Cigarette butts: A small but hazardous waste, according to European regulation. Waste Manag..

[92] Livazovic S., Li Z., Behzad A.R., Peinemann K.V., Nunes S.P. (2015). Cellulose multilayer membranes manufacture with ionic liquid. J. Membr. Sci..

[93] Ventura-Cruz S., Tecante A. (2021). Nanocellulose and microcrystalline cellulose from agricultural waste: Review on isolation and application as reinforcement in polymeric matrices. Food Hydrocoll..

[94] Lv J., Zhang G., Zhang H., Zhao C., Yang F. (2018). Improvement of antifouling performances for modified PVDF ultrafiltration membrane with hydrophilic cellulose nanocrystal. Appl. Surf. Sci..

[95] Iskandar M.J., Baharum A., Anuar F.H., Othaman R. (2018). Palm oil industry in South East Asia and the effluent treatment technology—A review. Environ. Technol. Innov..

[96] Teh K.C., Foo M.L., Ooi C.W., Leng Chew I.M. (2021). Sustainable and cost-effective approach for the synthesis of lignin-containing cellulose nanocrystals from oil palm empty fruit bunch. Chemosphere.

[97] Rasli S.R.A.M., Ahmad I., Lazim A.M., Hamzah A. (2017). Pengekstrakan dan pencirian selulosa daripada bahan buangan pertanian—Pelepah kelapa sawit. Malays. J. Anal. Sci..

[98] Shanmugarajah B., Chew I.M.L., Mubarak N.M., Choong T.S.Y., Yoo C.K., Tan K.W. (2019). Valorization of palm oil agro-waste into cellulose biosorbents for highly effective textile effluent remediation. J. Clean. Prod..

[99] Foo M.L., Ooi C.W., Tan K.W., Chew I.M.L. (2020). A Step Closer to Sustainable Industrial Production: Tailor the Properties of Nanocrystalline Cellulose from Oil Palm Empty Fruit Bunch. J. Environ. Chem. Eng..

[100] Liu W., Liu S., Liu T., Liu T., Zhang J., Liu H. (2019). Eco-friendly post-consumer cotton waste recycling for regenerated cellulose fibres. Carbohydr. Polym..

[101] Neelamegam A., Al-Battashi H., Al-Bahry S., Nallusamy S. (2018). Biorefinery production of poly-3-hydroxybutyrate using waste office paper hydrolysate as feedstock for microbial fermentation. J. Biotechnol..

[102] Xia G., Wan J., Zhang J., Zhang X., Xu L., Wu J., He J., Zhang J. (2016). Cellulose-based films prepared directly from waste newspapers via an ionic liquid. Carbohydr. Polym..

[103] Fan J., Zhang S., Li F., Yang Y., Du M. (2020). Recent advances in cellulose-based membranes for their sensing applications. Cellulose.

[104] Budtova T., Navard P. (2016). Cellulose in NaOH–water based solvents: A review. Cellulose.

[105] Klemm D., Heublein B., Fink H.P., Bohn A. (2005). Cellulose: Fascinating biopolymer and sustainable raw material. Angew. Chem.—Int. Ed..

[106] Shi Z., Liu Y., Xu H., Yang Q., Xiong C., Kuga S., Matsumoto Y. (2018). Facile dissolution of wood pulp in aqueous NaOH/urea solution by ball milling pretreatment. Ind. Crop. Prod..

[107] Chen X., Chen J., You T., Wang K., Xu F. (2015). Effects of polymorphs on dissolution of cellulose in NaOH/urea aqueous solution. Carbohydr. Polym..

[108] Lu F., Zhang C., Kang H., Huang Y., Liu R. (2016). Extensional rheology of cellulose/NaOH/urea/H2O solutions. Cellulose.

[109] Liu G., Li W., Chen L., Zhang X., Niu D., Chen Y., Yuan S., Bei Y., Zhu Q. (2020). Molecular dynamics studies on the aggregating behaviors of cellulose molecules in NaOH/urea aqueous solution. Colloids Surf. A Physicochem. Eng. Asp..

[110] Xiong B., Zhao P., Hu K., Zhang L., Cheng G. (2014). Dissolution of cellulose in aqueous NaOH/urea solution: Role of urea. Cellulose.

[111] Liu G., Sun H., Liu G., Zhang H., Yuan S., Zhu Q. (2018). A molecular dynamics study of cellulose inclusion complexes in NaOH/urea aqueous solution. Carbohydr. Polym..

[112] Isik M., Sardon H., Mecerreyes D. (2014). Ionic liquids and cellulose: Dissolution, chemical modification and preparation of new cellulosic materials. Int. J. Mol. Sci..

[113] Anokhina T.S., Pleshivtseva T.S., Ignatenko V.Y., Antonov S.V., Volkov A.V. (2017). Fabrication of composite nanofiltration membranes from cellulose solutions in an [Emim]OAc–DMSO mixture. Pet. Chem..

[114] Gericke M., Fardim P., Heinze T. (2012). Ionic liquids—Promising but challenging solvents for homogeneous derivatization of cellulose. Molecules.

[115] Uto T., Yamamoto K., Kadokawa J.I. (2018). Cellulose Crystal Dissolution in Imidazolium-Based Ionic Liquids: A Theoretical Study. J. Phys. Chem. B.

[116] Glińska K., Gitalt J., Torrens E., Plechkova N., Bengoa C. (2021). Extraction of cellulose from corn stover using designed ionic liquids with improved reusing capabilities. Process Saf. Environ. Prot..

[117] Kasavan S., Yusoff S., Rahmat Fakri M.F., Siron R. (2021). Plastic pollution in water ecosystems: A bibliometric analysis from 2000 to 2020. J. Clean. Prod..

[118] Geyer R., Jambeck J.R., Law K.L. (2017). Production, use, and fate of all plastics ever made. Sci. Adv..

[119] Li W.C., Tse H.F., Fok L. (2016). Plastic waste in the marine environment: A review of sources, occurrence and effects. Sci. Total. Environ..

[120] Kubowicz S., Booth A.M. (2017). Biodegradability of Plastics: Challenges and Misconceptions. Environ. Sci. Technol..

[121] Lin C.H., Gung C.H., Wu J.Y., Suen S.Y. (2015). Cationic dye adsorption using porous composite membrane prepared from plastic and plant wastes. J. Taiwan Inst. Chem. Eng..

[122] Li G., Wang J., Hou D., Bai Y., Liu H. (2015). Fabrication and performance of PET mesh enhanced cellulose acetate membranes for forward osmosis. J. Environ. Sci..

[123] Zander N.E., Gillan M., Sweetser D. (2016). Recycled PET nanofibers for water filtration applications. Materials.

[124] Ghazanfari D., Bastani D., Mousavi S.A. (2017). Preparation and characterization of poly (vinyl chloride) (PVC) based membrane for wastewater treatment. J. Water Process Eng..

[125] El-Gendi A., Abdallah H., Amin A., Amin S.K. (2017). Investigation of polyvinylchloride and cellulose acetate blend membranes for desalination. J. Mol. Struct..

[126] Demirel E., Zhang B., Papakyriakou M., Xia S., Chen Y. (2017). Fe_2_O_3_ nanocomposite PVC membrane with enhanced properties and separation performance. J. Membr. Sci..

[127] Garcia-Ivars J., Wang-Xu X., Iborra-Clar M.I. (2017). Application of post-consumer recycled high-impact polystyrene in the preparation of phase-inversion membranes for low-pressure membrane processes. Sep. Purif. Technol..

[128] Wu H., Li T., Liu B., Chen C., Wang S., Crittenden J.C. (2018). Blended PVC/PVC-g-PEGMA ultrafiltration membranes with enhanced performance and antifouling properties. Appl. Surf. Sci..

[129] Rajesh S., Murthy Z.V.P. (2014). Ultrafiltration membranes from waste polyethylene terephthalate and additives: Synthesis and characterization. Quim. Nova.

[130] Vaysizadeh A., Zinatizadeh A.A., Zinadini S. (2021). Fouling mitigation and enhanced dye rejection in UF and NF membranes via layer-by-layer (LBL) assembly and altering PVP percentage as pore former. Environ. Technol. Innov..

[131] Mamah S.C., Goh P.S., Ismail A.F., Suzaimi N.D., Ahmad N.A., Lee W.J. (2021). Flux enhancement in reverse osmosis membranes induced by synergistic effect of incorporated palygorskite/chitin hybrid nanomaterial. J. Environ. Chem. Eng..

[132] Fazli A., Rodrigue D. (2020). Waste rubber recycling: A review on the evolution and properties of thermoplastic elastomers. Materials.

[133] Bockstal L., Berchem T., Schmetz Q., Richel A. (2019). Devulcanisation and reclaiming of tires and rubber by physical and chemical processes: A review. J. Clean. Prod..

[134] Ma H., Shen J., Cao J., Wang D., Yue B., Mao Z., Wu W., Zhang H. (2017). Fabrication of wool keratin/polyethylene oxide nano-membrane from wool fabric waste. J. Clean. Prod..

[135] Ding J., Chen M., Chen W., He M., Zhou X., Yin G. (2018). Vapor-assisted crosslinking of a FK/PVA/PEO nanofiber membrane. Polymers.

[136] Zhong X., Li R., Wang Z., Wang W., Yu D. (2020). Eco-fabrication of antibacterial nanofibrous membrane with high moisture permeability from wasted wool fabrics. Waste Manag..

[137] David P.S., Karunanithi A., Fathima N.N. (2020). Improved filtration for dye removal using keratin–polyamide blend nanofibrous membranes. Environ. Sci. Pollut. Res..

[138] Karunanidhi A., David P.S., Fathima N.N. (2020). Electrospun Keratin-Polysulfone Blend Membranes for Treatment of Tannery Effluents. Water Air Soil Pollut..

[139] Teow Y.H., Amirudin S.N., Ho K.C. (2020). Sustainable approach to the synthesis of cellulose membrane from oil palm empty fruit bunch for dye wastewater treatment. J. Water Process Eng..

[140] Mohamed M.A., Salleh W.N.W., Jaafar J., Ismail A.F., Abd Mutalib M., Mohamad A.B., Zain M.F., Awang N.A., Mohd Hir Z.A. (2017). Physicochemical characterization of cellulose nanocrystal and nanoporous self-assembled CNC membrane derived from Ceiba pentandra. Carbohydr. Polym..

[141] Lopatina A., Anugwom I., Blot H., Sanchez Conde A., Manttari M., Kallioinen M. (2021). Re-use of waste cotton textile as an ultrafiltration membrane. J. Environ. Chem. Eng..

[142] Vignesh N., Suriyaraj S.P., Selvakumar R., Chandraraj K. (2021). Facile Fabrication and Characterization of Zn Loaded Cellulose Membrane from Cotton Microdust Waste and its Antibacterial Properties—A Waste to Value Approach. J. Polym. Environ..

[143] Mohamed M.A., Salleh W., Jaafar J., Ismail A.F., Mutalib M.A., Sani N.A.A., Asri S.E.A.M., Ong C.S. (2016). Physicochemical characteristic of regenerated cellulose/N-doped TiO_2_ nanocomposite membrane fabricated from recycled newspaper with photocatalytic activity under UV and visible light irradiation. Chem. Eng. J..

[144] Campano C., Miranda R., Merayo N., Negro C., Blanco A. (2017). Direct production of cellulose nanocrystals from old newspapers and recycled newsprint. Carbohydr. Polym..

[145] Rodrigues Filho G., Monteiro D.S., da Silva Meireles C., de Assunção R.M.N., Cerqueira D.A., Barud H.S., Ribeiro S.J.L., Messadeq Y. (2008). Synthesis and characterization of cellulose acetate produced from recycled newspaper. Carbohydr. Polym..

[146] Ünlü C.H. (2013). Carboxymethylcellulose from recycled newspaper in aqueous medium. Carbohydr. Polym..

[147] Mohamed M.A., Salleh W.N.W., Jaafar J., Ismail A.F., Abd Mutalib M., Jamil S.M. (2015). Feasibility of recycled newspaper as cellulose source for regenerated cellulose membrane fabrication. J. Appl. Polym. Sci..

[148] Liu W., Cui M., Shen Y., Zhu G., Luo L., Li M., Li J. (2019). Waste cigarette filter as nanofibrous membranes for on-demand immiscible oil/water mixtures and emulsions separation. J. Colloid Interface Sci..

[149] Doyan A., Leong C.L., Bilad M.R., Kurnia K.A., Susilawati S., Prayogi S., Narkkun T., Faungnawakij K. (2021). Cigarette butt waste as material for phase inverted membrane fabrication used for oil/water emulsion separation. Polymers.

[150] Aji M.M., Narendren S., Purkait M.K., Katiyar V. (2020). Utilization of waste polyvinyl chloride (PVC) for ultrafiltration membrane fabrication and its characterization. J. Environ. Chem. Eng..

[151] Mishra G., Mukhopadhyay M. (2018). Enhanced antifouling performance of halloysite nanotubes (HNTs) blended poly(vinyl chloride) (PVC/HNTs) ultrafiltration membranes: For water treatment. J. Ind. Eng. Chem..

[152] Aji M.M., Narendren S., Purkait M.K., Katiyar V. (2020). Biopolymer (gum arabic) incorporation in waste polyvinylchloride membrane for the enhancement of hydrophilicity and natural organic matter removal in water. J. Water Process Eng..

[153] Wang S.Y., Fang L.F., Cheng L., Jeon S., Kato N., Matsuyama H. (2018). Novel ultrafiltration membranes with excellent antifouling properties and chlorine resistance using a poly(vinyl chloride)-based copolymer. J. Membr. Sci..

[154] Fang L.-F., Jeon S., Kakihana Y., Kakehi J.-i., Zhu B.-K., Matsuyama H., Zhao S. (2017). Improved antifouling properties of polyvinyl chloride blend membranes by novel phosphate based-zwitterionic polymer additive. J. Membr. Sci..

[155] Adamczak M., Kamińska G., Bohdziewicz J. (2020). Application of waste polymers as basic material for ultrafiltration membranes preparation. Water.

[156] Kusumocahyo S.P., Ambani S.K., Kusumadewi S., Sutanto H., Widiputri D.I., Kartawiria I.S. (2020). Utilization of used polyethylene terephthalate (PET) bottles for the development of ultrafiltration membrane. J. Environ. Chem. Eng..

[157] Mulyati S., Armando M.A., Mawardi H., Fahrina A., Malahayati N., Muchtar S. (2018). Fabrication of hydrophilic and strong pet-based membrane from wasted plastic bottle. Rasayan J. Chem..

[158] Kiani S., Mousavi S.M., Bidaki A. (2021). Preparation of polyethylene terephthalate/xanthan nanofiltration membranes using recycled bottles for removal of diltiazem from aqueous solution. J. Clean. Prod..

[159] Dong L.X., Huang X.C., Wang Z., Yang Z., Wang X.M., Tang C.Y. (2016). A thin-film nanocomposite nanofiltration membrane prepared on a support with in situ embedded zeolite nanoparticles. Sep. Purif. Technol..

[160] Xu G.R., An X.C., Das R., Xu K., Xing Y.L., Hu Y.X. (2020). Application of electrospun nanofibrous amphiphobic membrane using low-cost poly (ethylene terephthalate) for robust membrane distillation. J. Water Process Eng..

[161] Doan H.N., Phong Vo P., Hayashi K., Kinashi K., Sakai W., Tsutsumi N. (2020). Recycled PET as a PDMS-Functionalized electrospun fibrous membrane for oil-water separation. J. Environ. Chem. Eng..

[162] Pulido B.A., Habboub O.S., Aristizabal S.L., Szekely G., Nunes S.P. (2019). Recycled Poly(ethylene terephthalate) for High Temperature Solvent Resistant Membranes. ACS Appl. Polym. Mater..

[163] Lin Y.T., Kao F.Y., Chen S.H., Wey M.Y., Tseng H.H. (2020). A facile approach from waste to resource: Reclaimed rubber-derived membrane for dye removal. J. Taiwan Inst. Chem. Eng..

[164] Gan L., Qiu F., Yue X., Chen Y., Xu J., Zhang T. (2021). Aramid nanofiber aerogel membrane extract from waste plastic for efficient separation of surfactant-stabilized oil-in-water emulsions. J. Environ. Chem. Eng..

[165] Nakasone K., Kobayashi T. (2016). Cytocompatible cellulose hydrogels containing trace lignin. Mater. Sci. Eng. C.

[166] Hamad K., Kaseem M., Deri F. (2013). Recycling of waste from polymer materials: An overview of the recent works. Polym. Degrad. Stab..

[167] Xie W., Li T., Tiraferri A., Drioli E., Figoli A., Crittenden J.C., Liu B. (2021). Toward the Next Generation of Sustainable Membranes from Green Chemistry Principles. ACS Sustain. Chem. Eng..

[168] Yadav P., Ismail N., Essalhi M., Tysklind M., Athanassiadis D., Tavajohi N. (2021). Assessment of the environmental impact of polymeric membrane production. J. Membr. Sci..

[169] Lee W.J., Goh P.S., Lau W.J., Ismail A.F., Hilal N. (2021). Green approaches for sustainable development of liquid separation membrane. Membranes.

[170] Gupta S.K., Mao Y. (2021). A review on molten salt synthesis of metal oxide nanomaterials: Status, opportunity, and challenge. Prog. Mater. Sci..

